# Prognostic significance of TMEM131L in glioma and establishment of oxidative stress prognostic model

**DOI:** 10.3389/fneur.2023.1162394

**Published:** 2023-04-06

**Authors:** Li Shan, Xiaoli Zhu, Hui-Zhu Qiu, Er-Dong Zuo, Xu Cheng

**Affiliations:** Department of Oncology, Soochow University Affiliated Taicang Hospital, (The First People’s Hospital of Taicang), Jiangsu, China

**Keywords:** glioma, TMEM131L, prognosis, oxidative stress, bioinformatics

## Abstract

Gliomas are the most aggressive of all brain tumors. In this study, it was found that there is a significant expression of transmembrane-like 131 (TMEM131L) in glioma tissues. The relevance of TMEM131L in the diagnosis and clinical prognosis of GBM and LGG was verified by additional clinical correlation and survival analysis. The area under the curve (AUC) of the receiver operating characteristic (ROC) curve reflected the diagnostic effect of TMEM131L on the clinicopathologic features of glioma. As a unique molecular marker for the poor prognosis of overall survival (OS), PFI, and DSS in patients with GCB and LGG, TMEM131L might be employed, according to time-dependent ROC curves and Kaplan–Meier survival analysis at 1, 3, and 5 years. The potential methylation sites of TMEM131L were selected by correlation analysis between TMEM131L and DNA methylation sites. Meanwhile, TMEM131L was significantly correlated with matrix, immunity, and estimated scores of GBM and LGG. The CIBERSORT analysis revealed a significant correlation between immune checkpoint and infiltration of 22 different kinds of immune cells. Coexpression genes of TMEM131L associated with oxidative stress phenotype were screened by the LASSO logistic regression analysis. Nomogram and calibration curves further confirmed that the prognostic model composed of SYT1, CREB3L3, ITPR1, RASGRF2, PDX1, and RASGRF1 has good stability and potential application value for poor prognosis in patients with glioma.

## Introduction

Gliomas are the most common primary tumors in the brain and spinal cord (1–3). Even with the availability of several therapies, glioma remains highly aggressive and lethal (4). In recent decades, specialized research in glioma biology has revealed some key genetic and molecular mechanisms (5). These findings contribute to a new understanding of glioma biology (4), change current classifications (6, 7), and provide new insights into tumorigenesis, ontogeny, and tumor progression (8). Recent studies have shown that regulators of TP53-induced glycolysis and apoptosis relieve oxidative stress and protect against ischemic neuronal damage after short-term ischemia, by converting glucose metabolism to pentose phosphate pathway (PPP). However, during prolonged ischemia, the brain changes glucose metabolism from PPP to glycolysis. The role of transmembrane-like 131 (TMEM131L) in exerting antioxidant activity and neuroprotective effects in the chronically ischemic brain is still unknown. In this study, the mechanisms of the impact of TMEM131L on the prognosis of patients with glioma and immune infiltration functions using the Cancer Genome Atlas (TCGA) database were initially assessed (9). Differentially expressed genes (DEGs) related to oxidative stress and prognosis were discovered. TMEM131L was identified as a new prognostic marker. Based on overall survival (OS) in both high and low TMEM131L expression groups, Kaplan–Meier (K-M) survival, time-dependent receiver operating characteristics (ROCs), and Cox regression analysis were used to assess the independent prognostic value of the clinical features of glioma. The oxidative stress-related coexpressed genes impacted by TMEM131L were further screened, and lasopenalized Cox regression analysis was used to create the predictive feature model. Analysis revealed several important coexpressed genes associated with prognosis, suggesting a potential biological mechanism through which TMEM131L may alter the prognosis of patients with glioma. Finally, the key genes were enriched by Gene Ontology (GO) and the Kyoto Encyclopedia of Genes and Genomes (KEGG).

## Materials and methods

### Data sources and collection

TCGA TARGET GTEx (PANCAN, *N* = 19,131, G = 60,499) was downloaded from the UCSC^1^ database, and further we extracted ENSG00000117650 (TMEM131L) gene expression data in each sample. We also eliminated cancer species with fewer than three samples in a single cancer species, resulting in 34 cancer species with expression data.

### Expression difference and correlation analysis of pan-cancer

The UCSC (see text footnote 1) database was used to download and standardize the pan-cancer dataset from which we extracted expression data for TMEM131L gene. Transcriptomics information (read) and clinical information from the TCGA,^3^ including patient age, gender, stage, and overall survival rate, are both available. This study contained 703 samples in all. The “limma” package was used to normalize the initial expression data, and genes whose average expression was less than 1 were eliminated (10). The “limma” software was additionally utilized for MTG differential expression analysis. To find differentially expressed genes of standard for |logFC| < 1 and adj.P < 0.05 in this study. The read count was converted to a TPM value, and since the TPM value was the same as the microarray value, the log2 + 1 conversion was performed for further analysis. The sample set was split into k identically sized, mutually exclusive subgroups using the K-fold cross-validation method, each of which attempted to preserve the consistency of the data distribution.

### Evaluation of patient characteristics

We identified TMEM131L’s diagnostic value for GBM in TCGA based on its expression. To assess biomarkers for predicting patient survival, ROC curves were produced (11). The nomogram survival probability plot can be accessed using “rms” and “foreign” based on multivariate logistic regression. Additionally, we compared the nomogram and Kaplan–Meier survival probabilities and evaluated the concordance index (c-index) (12).

### TMEM131L gene set enrichment analysis

To establish the statistical significance of a collection of genes that were chosen with preference and whether there were consistent differences between the two biological states, we utilized the gene set enrichment analysis (GSEA). In this study, we permuted each gene combination analyzed a thousand times. We used TMEM131L expression levels as phenotypic markers. The gene set “c2.cp.v7.2.symbols.gmt” was retrieved from a molecular marker database; potential enrichment pathways were analyzed with GSEA 4.0.3. We also generated normalized enrichment scores (NES), nom value of *p*s, and FDR *q* values for GSEA to separate enrichment pathways. Statistically significant gene sets were defined as those with nom value of *p* > 0.05 and FDR value of *q* > 0.25.

### Development and validation of a risk prediction model for oxidative stress

Standard operating procedures that have been defined for data processing are also created in order to facilitate data transfer. Combined with TCGA clinical data, the risk prediction model of oxidative stress-related TMEM131L co-expression gene was constructed. The prognostic related hub gene was further analyzed using the least absolute shrinkage and selection operator (LASSO) technique for dimensional reduction analysis and feature selection. Based on the LASSO regression coefficients, we computed the risk scoring algorithm.


$$\mathrm{risk}Score=\underset{i}{\sum }Coefficient\;\left(hub\;gene_{i}\right)*mRNAExpression\;\left(hub\;gene_{i}\right)$$

### Immune infiltration

“Estimate” package was used to analyze the purity of 33 human cancers, based on immune and stromal score (13). Indirectly indicating tumor purity, the ESTIMATE score was the total of earlier scores. The relationships between TMEM131L expression and ESTIMATE scores in various malignancies were shown in the scatter plots as the total of earlier scores. A comprehensive tool for the systematic investigation of immune infiltrates in various cancer types is TIMER2.0.^4^ First, the expression of TMEM131L in tumors and nearby normal tissues was compared in all TCGA cohorts. The next step was to use several immune deconvolution algorithms to examine the connection between TMEM131L expression and immune infiltration (14, 15). In order to determine if immunotherapy responders and non-responders had significantly different levels of TMEM131L expression, we also used TISIDB^5^ (16). Finally, we investigated the relationship between TMEM131L and immune cell subsets.

### Analyses of the relative quantity of immune cells that infiltrate tumors

TIICs and TMEM131L expression were evaluated for their relative correlation using CIBERSORT^6^ (17). This technique can be used to characterize complex tissues. The association between the TMEM131L high and low expression groups was evaluated by the immunological responses of 22 TIICs using the CIBERSORT technique. The value of *p* for each sample is then determined using a deconvolution technique.

### Cell culture and transient transfection

U87 and U251 cells were purchased from ATCC (Manassas, United States). Cells were cultured in Gibco DEME F-12 medium (Thermo Fisher, United States). TMEM131L siRNA (Invitrogen, United States) and the negative control (NC) were transfected into the cells using Lipofectamine 2000 (Invitrogen, United States). The target sequences for TMEM131L siRNAs were CAGAGCTTCTCGGACAAACTATTTA (TMEM131L-si-1) and TAGCACATTGTGGCATGCATTATTT (TMEM131L-si-2).

### Real-time PCR

Utilizing the Total RNA kit to extract total RNA from grown cells (Vazyme, China). Using the Prime Script RT Reagent Kit, reverse transcription was carried out (Takara, Japan). The HiScript II One Step RT-PCR Kit was used to perform the reverse transcription-polymerase chain reaction (Vazyme, China). All primers were obtained from Sangon Biotech (Suzhou, China). The primer sequences are:

**Table:** 

Gene	Forward primer (5–3)	Reverse primer (5–3)
TMEM131L	CAGTTTACCTGCTGCCCAGA	CATACACATCGCTTTGCGGG
SYT1	AACAGCATGATGAGCCTCCG	CATCCTTGAGGGCCTGATCT
CREB3L3	ATCCTGGCAACTCTTGCTCC	AGGTGATGCTGTTGCAGGTC
ITPR1	AGTTTCAGCCCTCAGTGGAC	TCAGCAGGAGAAACCGGAAC
RASGRF1	GACATCAGCCAGTGTGTGGA	CTGGCAGGAATGGCACTGAT
RASGRF2	GTTAGGGCGGAGAGCGTG	CGCATGAAGCTCTGAACCTTT
PDX1	CCAGTGGGCAGGCGG	AGCCACAAACAACGCCAATC
GAPDH	AATGGGCAGCCGTTAGGAAA	GCCCAATACGACCAAATCAGAG

### Immunohistochemistry staining

Following deparaffinization and dehydration, tissue sections underwent epitope retrieval, non-specific binding blocking, and H2O2 treatment. Following that, slices were treated at 4°C for an overnight incubation with rabbit anti-human TMEM131L antibodies (1:100, Novus, NBP2-30898). The tissue samples were then treated for 1.5 h at room temperature with secondary antibodies (1:100, Proteintech, SA00001-2). An improved DAB staining kit was used to detect the signal (Proteintech).

### CCK8 assay

Cells were seeded into a 96-well plate at a density of 1,000 cells per well. CCK8 reagent (Beyotime, China) was added into the wells and cells cultured for 1.5 h. The absorbance was determined at 450 nm.

### Flow cytometry

The cell cycle of U87 and U251 was analyzed by flow cytometry according to the manufacturer’s protocol. To detect the cell cycle, cells were harvested with trypsin and then resuspended in PBS at a concentration of 1 × 10(5)/100 μl. Cells were then stained with PI on ice for 30 min. After washing with PBS, samples were detected by BD FACS Calibur flow cytometry (United States). Cell cycle distribution was analyzed by FlowJo software.

### Wound healing and transwell assay

In six-well plates, cell monolayers were seeded and scraped with a sterile 200 L pipette tip. In order to test for invasion, cells [5 × 10 (4)] were injected into either Matrigel-coated or Matrigel-uncoated chambers (for migration). The top layer was supplemented with SFM, and the bottom layer was introduced to a DMEM-only medium. Cells were stained with 0.1% crystalline violet after 24 h of incubation, and they were then counted under a light microscope.

### Statistical analysis

Statistical analysis was performed using R software (v.3.6.1). The cutoff value was decided upon as being the median TMEM131L expression. By using the chi-PCR technique, chisquare, and Fisher exact tests, the clinicopathological features of the TMEM131L high and low expression groups were examined. Using the Wilcoxon signed-rank test, the expression of TMEM131L was also linked with clinicopathological variables. Multiple group comparisons were made using the Kruskal-Wallis test. We used Cox regression and the Kaplan–Meier technique to evaluate overall survival (OS) in TCGA patients in order to evaluate the impact of TMEM131L expression on survival and clinicopathological variables. *p* < 0.05 was considered significant.

## Results

### Differential expression of TMEM131L in pan-cancer and its prognostic significance

**Figure 1:**
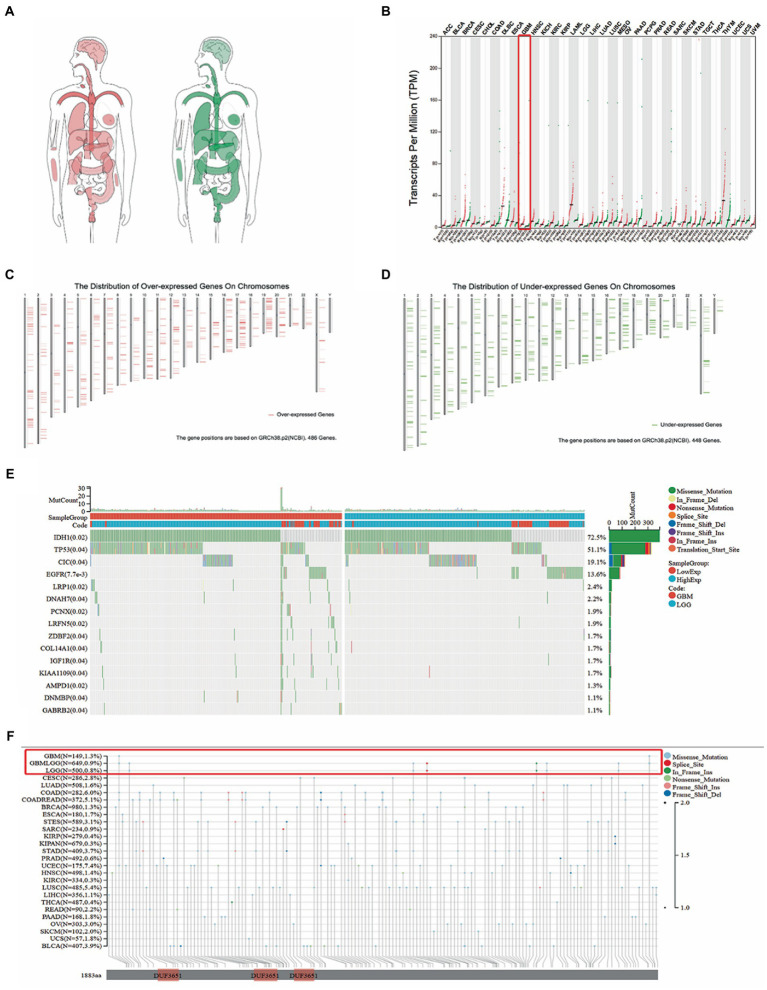
The expression of TMEM131L was analyzed by GEPIA database. **(A)** The expression of TMEM131L varies between tumor tissues (red) and normal tissues (green); **(B)** Differential expression map between tumor tissue and normal samples in pan-carcinoma; **(C,D)** Comparison of the position of mutations in each chromosome in glioma; **(E)** Top15 TMEM131L high and low expression related in TCGA-LGG data set, and gene mutation waterfall with significant difference in mutation frequency; **(F)** Pan-carcinoma analysis of TMEM131L mutation types.

Based on the Gene Expression Profiling Interactive Analysis (GEPIA) database, the difference in TMEM131L expression in human organs between normal tissues (green) and disease tissues (red) was initially analyzed. It was found that TMEM131L may be highly expressed in brain tumor tissues (Figure 1A). The pan-carcinoma expression differential map subsequently revealed that elevated expression of TMEM131L may be a sign of GBM (Figure 1B). Meanwhile, the high and low expression of chromosome mutant genes in glioma were compared, and it was observed that glioma did not have any notable high or low expression mutation (Figures 1C,D). Subsequently, the top 15 significantly related mutated genes of TMEM131L in different types of gliomas with gene mutation waterfall diagram were revealed. IDH1, TP53, and CIC mutations were more common in LGG among groups with high and low expression of TMEM131L, while EGFR mutations were more common in GBM (Figure 1E). Meanwhile, comparing various types of gene mutations in pan-carcinoma revealed that GBM and LGG mostly had gene deletion mutations (Figure 1F).

**Figure 2:**
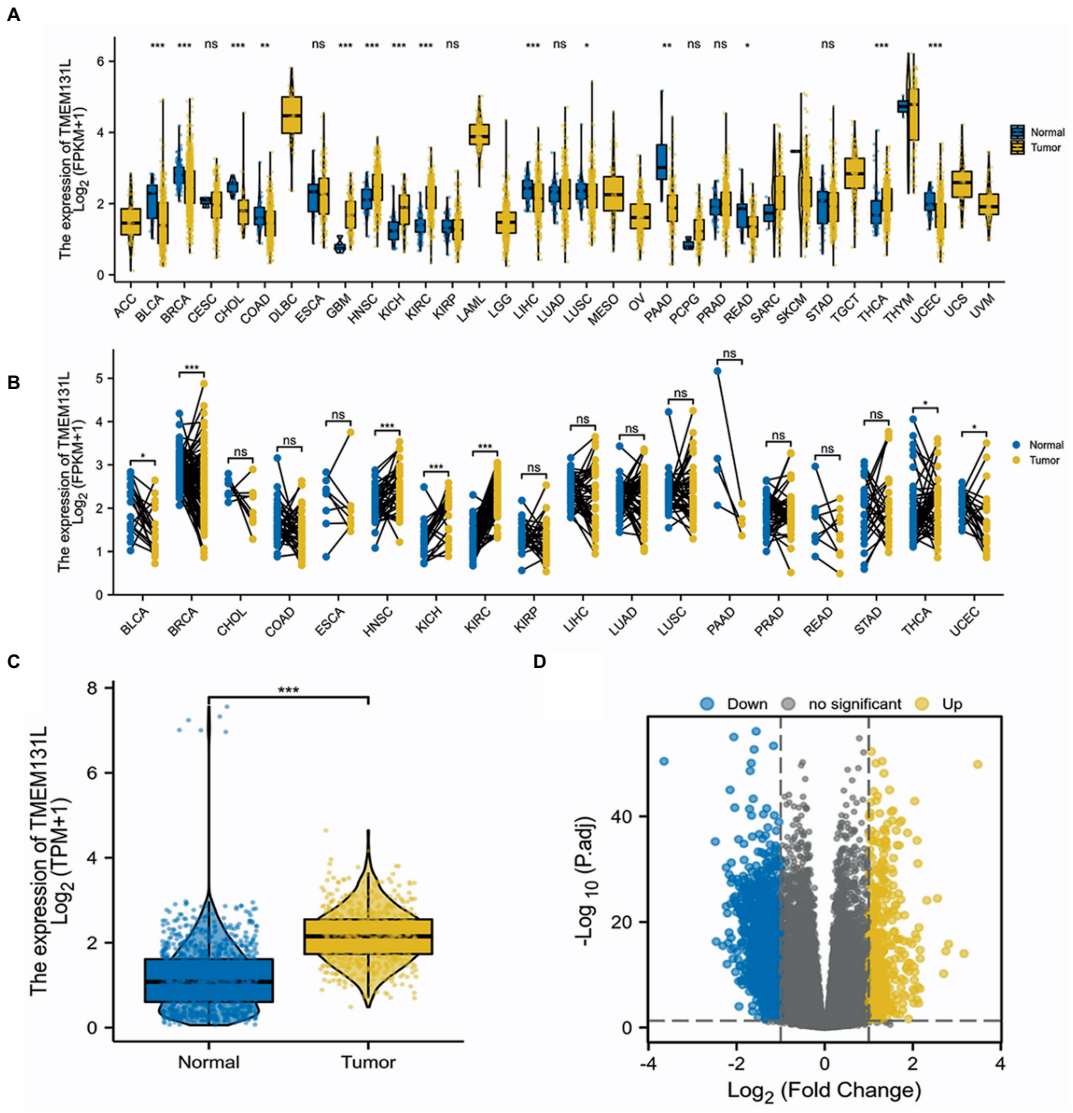
Differences in pan-carcinoma expression of TMEM131L. **(A,B)** Expression difference of TMEM131L in non-tumor tissues and unpaired and paired samples in the TCGA and GTEx pan-carcinoma database; **(C)** Expression difference of TMEM131L in TCGA and GTEx GBMLGG cohort; **(D)** Volcanic map of TMEM131L coexpression gene differences in TCGA and GTEx GBMLGG cohort.

The identification of genetic mutations in tumors is critical for the prognosis of tumors. This study was carried out to understand the role of TMEM131L mutations in pan-cancer, especially in GBM and LGG. The unpaired Wilcoxon Rank Sum and Signed Rank Tests were utilized to carry out the difference significance analysis between pennies, and the Kruskal test was conducted to carry out the difference test between multiple sets of samples. The differential expression of TMEM131L in pan-carcinoma was analyzed. Analysis of differential expression of TMEM131L between nontumor tissues and unpaired and paired samples of tumor tissues in TCGA and the GTEx pancarcinoma database indicated that differential expression of TMEM131L was significant in most tumors, including glioma (Figures 2A,B). Moreover, a significantly high expression of TMEM131L was observed in tumor tissues of the GBMLGG cohort (Figure 2C). The coexpression gene of TMEM131L in the GBMLGG samples was also demonstrated. 0.05 and |log2FC| and gt; (1) Differential volcano map (Figure 2D).

### The similarity of glioblastoma to other types of cancer

Numerous studies have shown that glioblastoma development, progression, and treatment resistance are significantly influenced by oxidative stress-related pathways. Therefore, the association of TMEM131L with STEMNESS markers and genes critical for oxidative stress was analyzed. Molecular markers that were significantly associated with glioma progression and oxidative stress pathways were found. Tetraspanin CD151 has been proven to be able to control the growth of glioblastoma tumor cells in glioma studies. Notably, PHF20 was originally found to be a tumor-specific antigen in GBM. Patients treated with antibodies to PHF20 were significantly better than those not treated with antibodies. Changes in metabolism in IDH mutant gliomas affect pathways like phospholipid, energy, and oxidative stress. Additionally, cancers have stem cell characteristics, such as self-renewal, multilineage differentiation, and possible resistance to standard treatments (18). The glioma situation is affected by the dryness of tumor cells. According to several studies, SOX2 is highly expressed in the GSC subgroup. Other studies have confirmed that nonadherent glioblastoma cells can promote SARBOX-2 expression and reactive oxygen species (ROS) accumulation. Based on previous research, high-grade astrocytoma exhibits much lower levels of GFAP expression than low-grade astrocytoma. According to another prognostic analysis, GFAP expression and MGMT promoter methylation were linked to the survival prognosis of patients with glioma.

**Figure 3:**
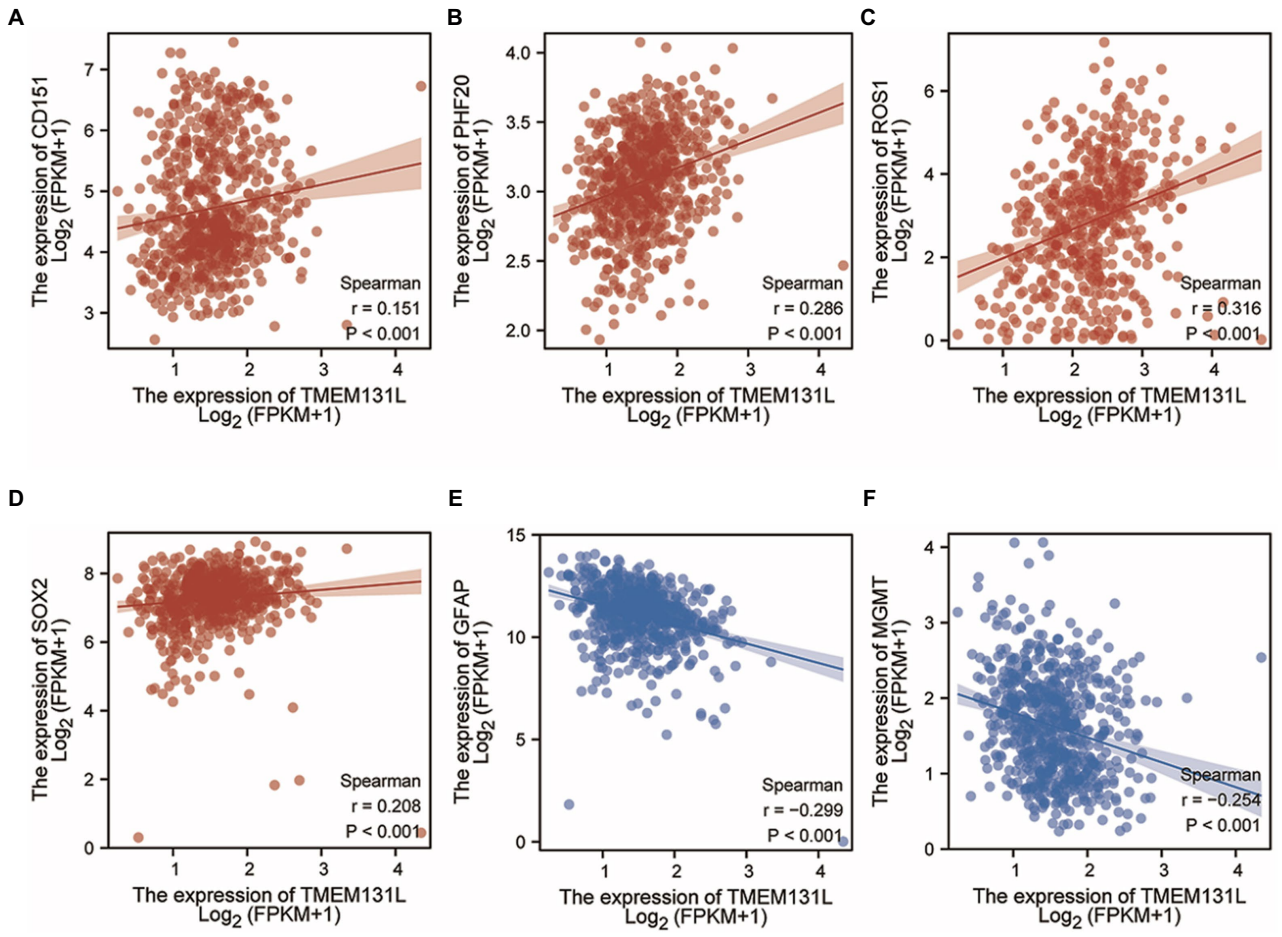
Correlation between TMEM131L and molecular markers of GBM and oxidative stress. **(A–D)** TMEM131L was positively correlated with the expressions of CD151, PHF20, ROS1, and SOX2, respectively. **(E, F)** TMEM131L was negatively correlated with GFAP and MGMT expression, respectively.

Correlation analysis showed that TMEM131L was positively correlated with the expression of CD151, PHF20, ROS1, and SOX2 (Figures 3A–D) and negatively correlated with the expression of GFAP and MGMT (Figures 3E–F). The above results suggest that TMEM131L may affect the occurrence of glioma by participating in oxidative stress-related molecular pathways.

### Diagnostic efficacy of TMEM131L for GBM and LGG and differential efficacy of clinical variables

**Figure 4:**
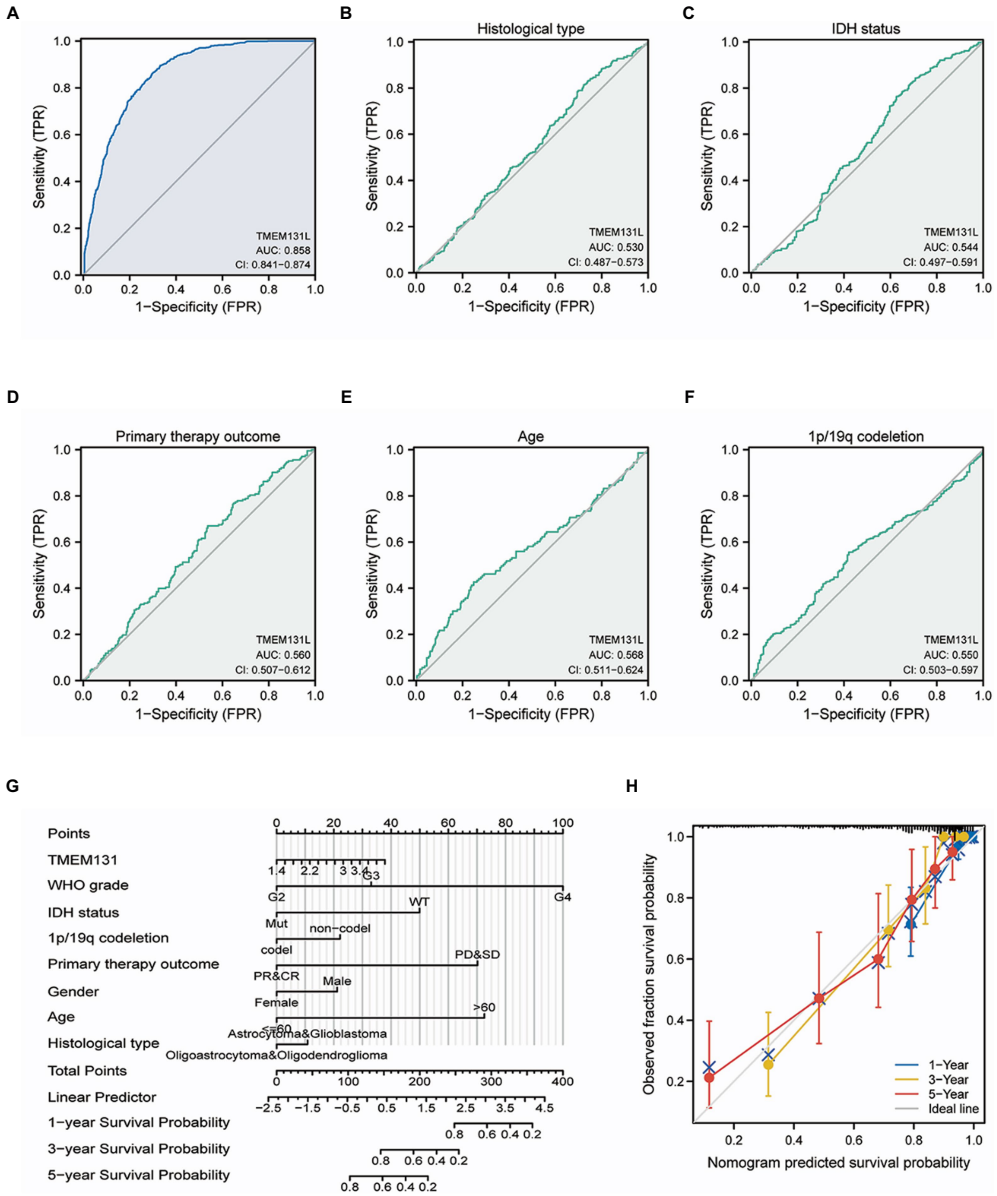
Differential efficacy of TMEM131L for the diagnosis and clinical variables of GBM and LGG. **(A)** Analysis of the diagnostic efficacy of TMEM131L for GBM and LGG. **(B–F)** Diagnostic efficacy of TMEM131L on Histological type, IDH status, Primary therapy outcom, Age, and 1p/19q codeletion; **(G,H)** Nomogram and calibration curve of TMEM131L and clinical variables.

Transmembrane-like 131 showed high diagnostic efficacy for GBM and LGG, with area under the curve (AUC): 0.858 and confidence interval (CI): 0.841–0.874 (Figure 4A). For histological type and IDH status, the AUC and CI of TMEM131L were 0.530 and 0.487–0.573, respectively, indicating a low diagnostic efficiency. An AUC value of 0.544 and CI of 0.497–0.59 indicates low diagnostic efficiency. In comparison, the AUC and CI of TMEM131L for the primary therapy outcome were 0.560 and 0.507–0.612, respectively, suggesting low diagnostic efficiency. Age, AUC: 0.568, CI: 0.511–0.624, and 1p/19q codeletion (AUC: 0.550; CI: 0.503–0.597) for low diagnostic efficacy by analysis of TMEM131L (Figures 4B–F).

**Figure 5:**
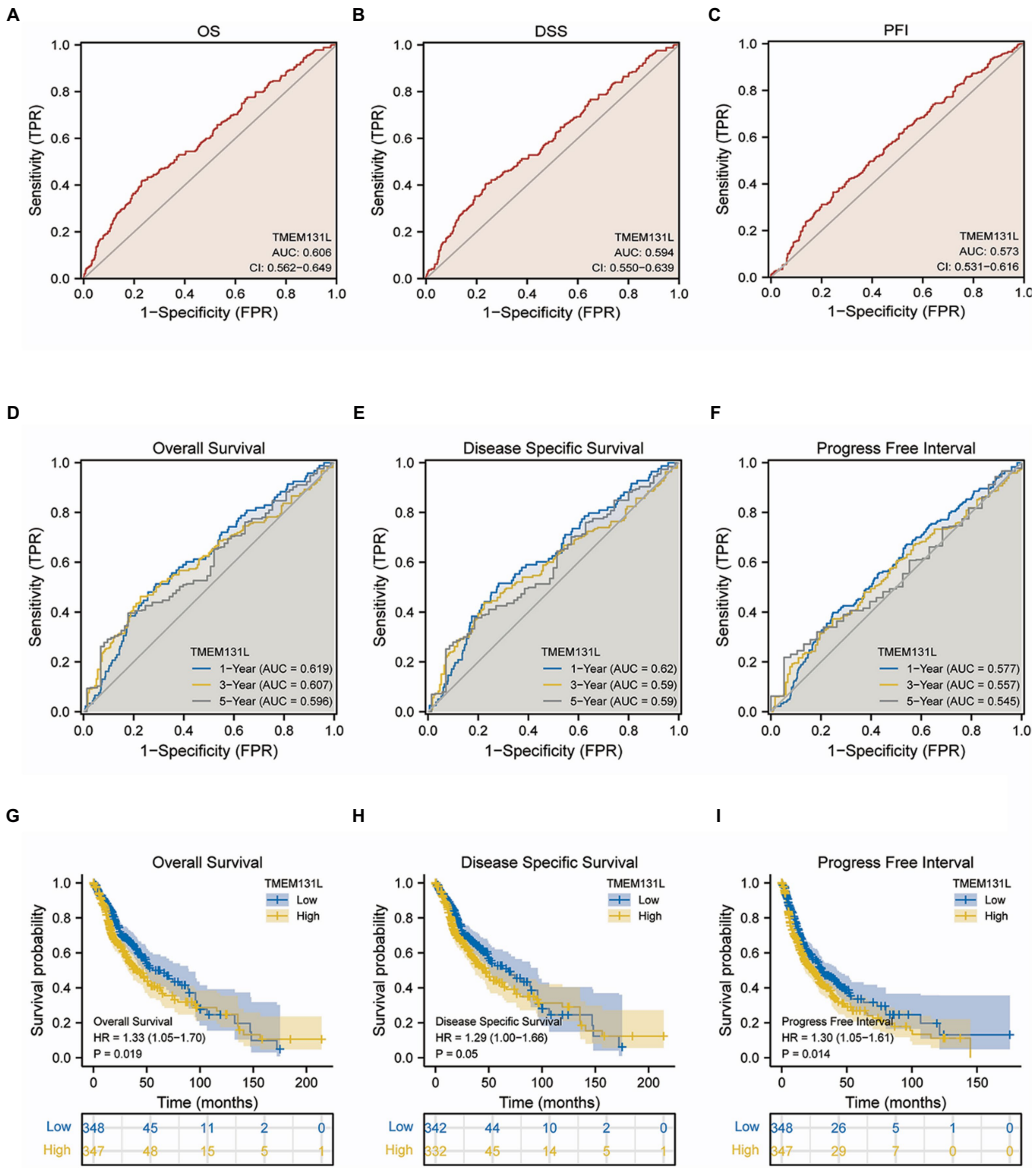
Identification and survival analysis of TMEM131L for poor prognosis of OS, PFI, and DSS in GCB and LGG patients. **(A–C)** Differential survival of TMEM131L in OS, PFI, and DSS in GCB&LGG patients; **(D–F)** Time-dependent ROC curves of TMEM131L for 1, 3, and 5-year OS, DSS, and PFI in patients with GCB&LGG; **(G–I)** Kaplan–Meier survival analysis curve of different survival status of GCB&LGG patients with OS, PFI and DSS based on the expression of TMEM131L.

The prediction efficiency of TMEM131L for survival status was also statistically significant, with OS, DSS, and PFI having the following values of values: AUC: 0.606 and CI: 0.562–0.649; AUC: 0.594 and CI: 0.550–0.639; and AUC: 0.573 and CI: 0.531–0.616, respectively (Figures 5A–C). Further the prognostic efficacy of TMEM131L in 1, 3, and 5-year survival was analyzed. The AUC values of 1-, 3-, and 5-year survival of OS based on GBM and LGG were 0.619, 0.607, and 0.596, respectively. The AUC values of 1-, 3-, and 5-year survival of DSS were 0.62, 0.59, and 0.59, respectively; 1-year (AUC = 0.577), 3-year (AUC = 0.557), and 5-year (AUC = 0.545; Figures 5D–F).

### Survival and clinical variables of TMEM131L in GBM and LGG

Survival analysis found that the following set of values was observed for OS, disease-specific survival, and progress-free interval, respectively: hazard ratio (HR) = 1.33 (1.05–1.70); *p* = 0.019; HR = 1.29 (1.00–1.66); *p* = 0.05; and HR = 1.30 (1.05–1.61); *p* = 0.014 (Figures 5G–I). To further analyze the prognostic ability of TMEM131L in patients with GCB and patients with LGG, a variety of clinicopathological variables that affected the prognosis of those patients, including the World Health Organization (WHO) grade, one-treatment outcome, IDH status, gender, and age, were collaboratively included. The predictive value of TMEM131L for clinical outcome variables in patients with GBM and LGG was visualized using a column graph. The contribution of the above prognostic factors to outcome variables was analyzed, their value levels were scored, and the total score was obtained by adding the scores. Therefore, the significance of the multi-index combination in predicting the risk or prognosis of GBM and LGG was obtained. Subsequently, the calibration curve was analyzed for verification (Figures 4G,H).

### The difference in expression of TMEM131L in clinical variables of GCB and LGG

Based on clinicopathologic data from GCB and LGG patient samples in TCGA database, the variation in clinical baseline between the TMEM131L high-expression and low-expression groups was examined. The significant clinical variables of TMEM131L expression affecting the prognosis of GCB and LGG were WHO grade (*p* = 0.001), IDH status (*p* = 0.328), 1p/19q co-deletion (*p* = 0.011), primary therapy result (*p* = 0.024), and histological type (p = 0.001). The difference in TMEM131L expression was significant in OS (*p* = 0.007), DSS (*p* = 0.024), and PFI (*p* = 0.012; Tables 1, 2).

**Table 1:** Baseline analysis of clinicopathological data between TMEM131L high and low expression groups in the TCGA-GCBLGG dataset.

Characteristic	Levels	Low expression of TMEM131L	High expression of TMEM131L	*p*
*n*		348	348	
WHO grade, *n* (%)	G2	135 (21.3%)	89 (14%)	**< 0.001**
	G3	116 (18.3%)	127 (20%)	
	G4	67 (10.6%)	101 (15.9%)	
IDH status, *n* (%)	WT	116 (16.9%)	130 (19%)	0.328
	Mut	226 (32.9%)	214 (31.2%)	
1p/19q codeletion, *n* (%)	codel	71 (10.3%)	100 (14.5%)	**0.011**
	non-codel	275 (39.9%)	243 (35.3%)	
Primary therapy outcome, *n* (%)	PD	51 (11%)	61 (13.2%)	**0.024**
	SD	77 (16.7%)	70 (15.2%)	
	PR	32 (6.9%)	32 (6.9%)	
	CR	89 (19.3%)	50 (10.8%)	
Histological type, *n* (%)	Astrocytoma	110 (15.8%)	85 (12.2%)	**< 0.001**
	Glioblastoma	67 (9.6%)	101 (14.5%)	
	Oligoastrocytoma	86 (12.4%)	48 (6.9%)	
	Oligodendroglioma	85 (12.2%)	114 (16.4%)	
OS event, *n* (%)	Alive	230 (33%)	194 (27.9%)	**0.007**
	Dead	118 (17%)	154 (22.1%)	
DSS event, *n* (%)	Alive	233 (34.5%)	198 (29.3%)	**0.024**
	Dead	109 (16.1%)	135 (20%)	
PFI event, *n* (%)	Alive	192 (27.6%)	158 (22.7%)	**0.012**
	Dead	156 (22.4%)	190 (27.3%)	
Age, *n* (%)	<=60	287 (41.2%)	266 (38.2%)	0.061
	>60	61 (8.8%)	82 (11.8%)	
Race, *n* (%)	Asian	5 (0.7%)	8 (1.2%)	0.616
	Black or African American	18 (2.6%)	15 (2.2%)	
	White	316 (46.3%)	321 (47%)	
Gender, *n* (%)	Female	148 (21.3%)	150 (21.6%)	0.939
	Male	200 (28.7%)	198 (28.4%)	

**Table 2:** Multivariate logistic regression of TMEM131L expression on prognosis of overall survival in GCB and LGG.

Characteristics	Total (N)	Odds ratio (OR)	*p* value
WHO grade (G3&G4 vs. G2)	635	1.890 (1.360–2.636)	<0.001
1p/19q codeletion (Non-codel vs. codel)	689	0.627 (0.441–0.889)	0.009
Primary therapy outcome (PR and CR vs. PD and SD)	462	0.662 (0.456–0.959)	0.030
IDH status (Mut vs. WT)	686	0.845 (0.618–1.155)	0.290
Gender (Male vs. Female)	696	0.977 (0.723–1.319)	0.878
Histological type (Oligoastrocytoma and Oligodendroglioma vs. Astrocytoma and Glioblastoma)	696	0.902 (0.669–1.214)	0.495
Age (>60 vs. <=60)	696	1.450 (1.002–2.108)	0.049

**Figure 6:**
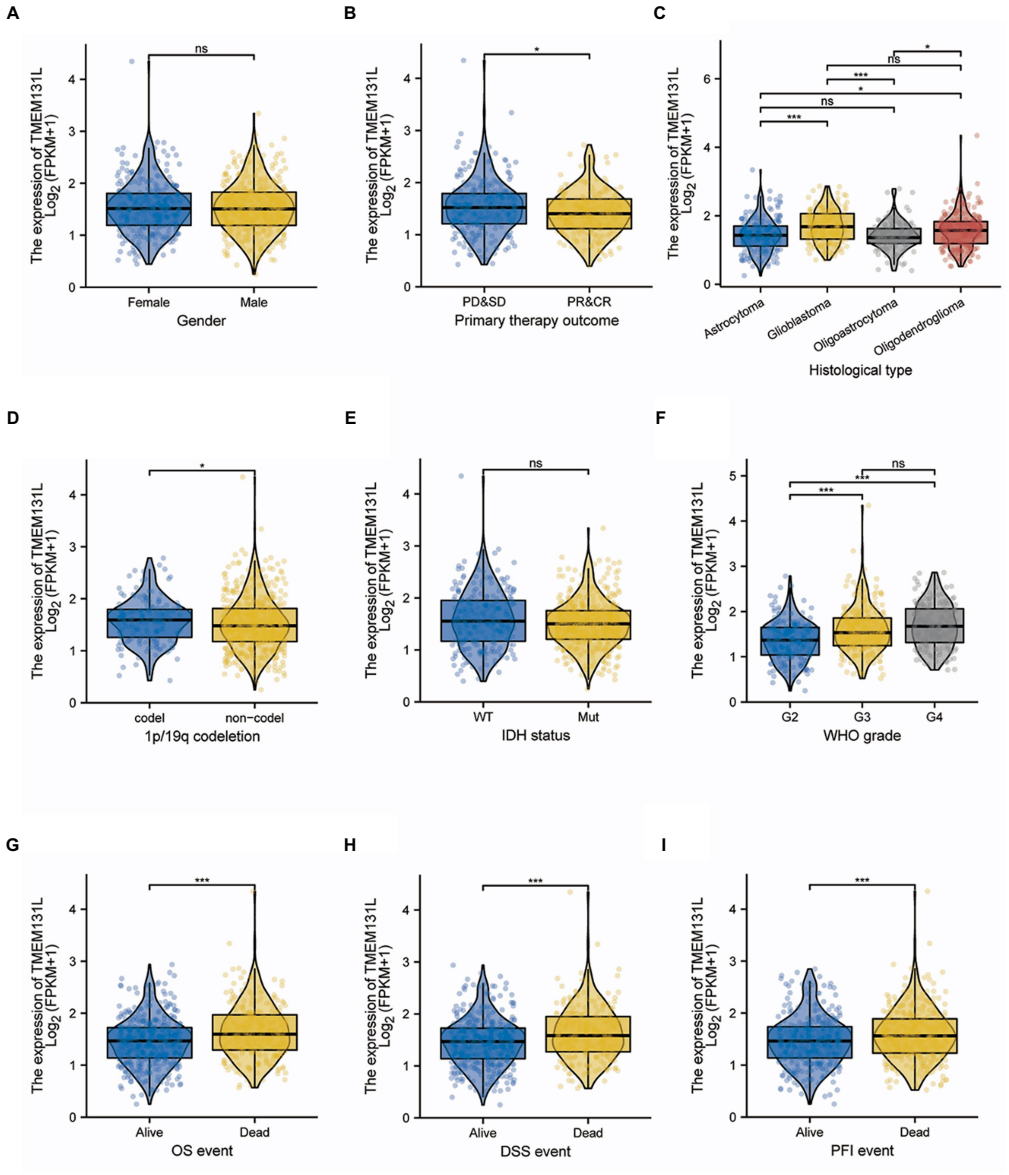
Correlation between TMEM131L expression and clinicopathological features in the TCGA-GBMLGG dataset. **(A–F)** Differences in expression of pathological parameters between subgroups; **(G–I)** Expression difference of TMEM131L in GBM and LGG patients under different OS, PFI, and DSS survival states.

The differences in the expression of TMEM131L among different pathological variable subgroups were further analyzed. High expression of TMEM131L did not significantly correlate with gender or IDH status. However, for noncoding subgroups with 1p/19q codeletion, poorer WHO scores were significantly associated with the histological type (Figures 6A–F). The findings revealed a substantial relationship between the high expression of TMEM131L and WHO grade, primary therapeutic outcome, and poor prognosis of the 1p/19q codeletion (Figures 6B,D,F). Meanwhile, the high expression of TMEM131L in histological subtypes of glioblastoma and oligodendroglioma was significant (Figure 6C). In comparison, TMEM131L was more significant in the identification of prognostic survival status of children with glioma. TMEM131L was still significantly expressed in the subgroup with poor prognosis even though there was no statistically significant difference between the high-and low-expression groups in gender and IDH status (Figures 6A,E). These results suggested that high expression of TMEM131L indicated a poor prognosis of glioma.

**Figure 7:**
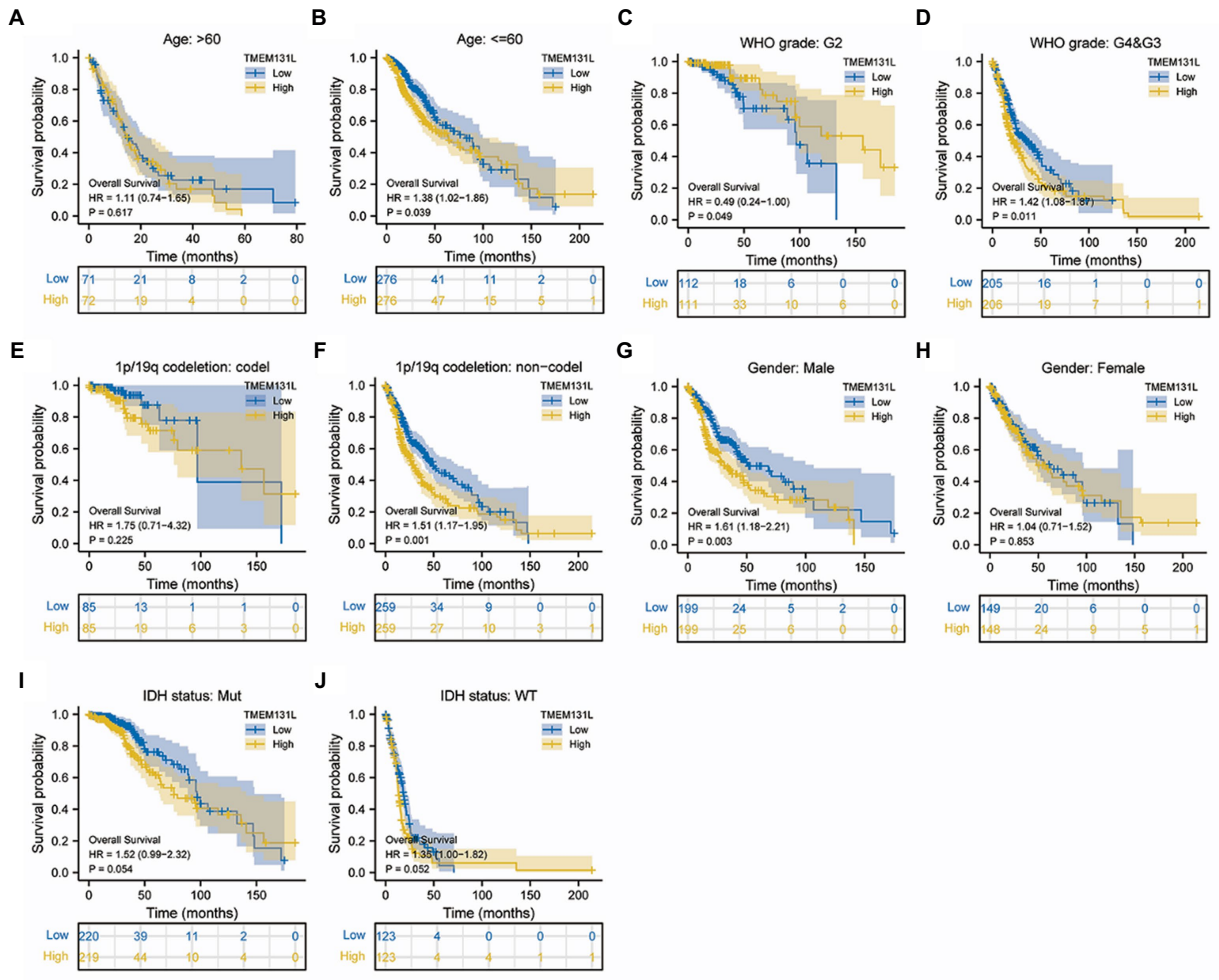
In TCGA-GBMLGG, the K-M curve of TMEM131L affected the overall survival of GBM and LGG patients in each pathological subgroup. TMEM131L had an overall survival prognostic significance in age **(A,B)**, WHO grade **(C,D)**, 1p/19q codeletion: codel **(E,F)**, Gender **(G,H)**, and IDH status **(I,J)** pathological subgroups, respectively.

Transmembrane-like 131 was considerably overexpressed in patients with GCB and LGG with OS (alive vs. dead), PFI (alive vs. dead), and DSS (alive vs. dead) clinical subgroups with poor prognosis, according to subgroup differential expression analysis of TMEM131L clinical factors (Figures 6G,H,I). To further analyze the OS prognostic power of TMEM131L in patients with GBM and LGG, the effect of differential expression of TMEM131L in the subgroups of various pathological variables of GBM and LGG was analyzed using K-M curves. The results suggested an OS prognostic significance in age (Figures 7A,B), WHO grade (Figures 7C,D), 1P/19q Codel (Figures 7E,F), sex (Figures 7G,H), and IDH status (Figures 7I,J) pathological subgroups.

### DNA methylation of TMEM131L

**Figure 8:**
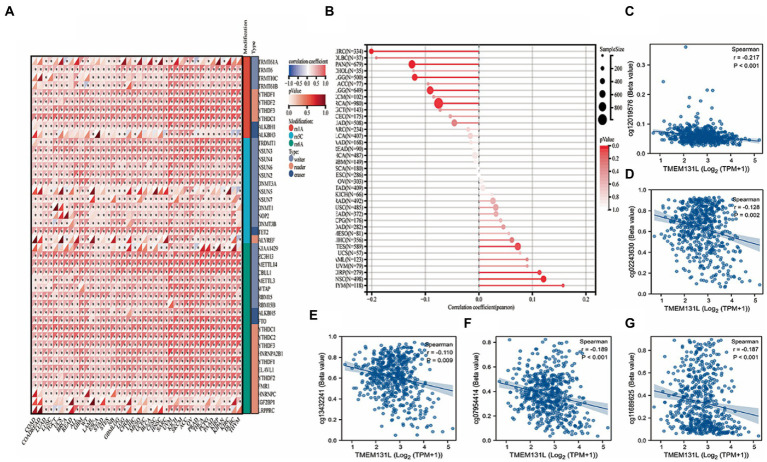
DNA methylation analysis of TMEM131L. **(A)** The correlation between TMEM131L and various key genes of RNA modification [m1A (10), m5C (13), m6A (21)] was shown by relevant heat maps; **(B)** The degree of methylation of TMEM131L in pan-carcinoma; **(C–G)** Scatter plot of TMEM131L with significant correlation with each methylation site.

The relationship between TMEM131L and several important genes was examined to determine whether the expression of TMEM131L in pan-carcinomas impacts the molecular mechanisms of cancer. Initially, 44 marker genes of three RNA-modified [m1A (10), m5C (13), and m6A (21)] genes (Figure 8A) were investigated, and the extent of methylation of TMEM131L in pan-cancerous tissues (Figure 8B) was analyzed. Meanwhile, their expression in each sample was partially significantly correlated with greater TMEM131L. Theoretically, the expression and methylation profiles of colocalization pairs were more negatively correlated than positively correlated (55% were negatively correlated). Also largely consistent was the correlation study between the expression of TMEM131L and certain methylation sites. The substantial link between TMEM131L and each methylation site is represented by a scatter plot (Figures 8C–G).

### Correlation analysis of ESTIMATE pan-cancer immune score based on TMEM131L

**Figure 9:**
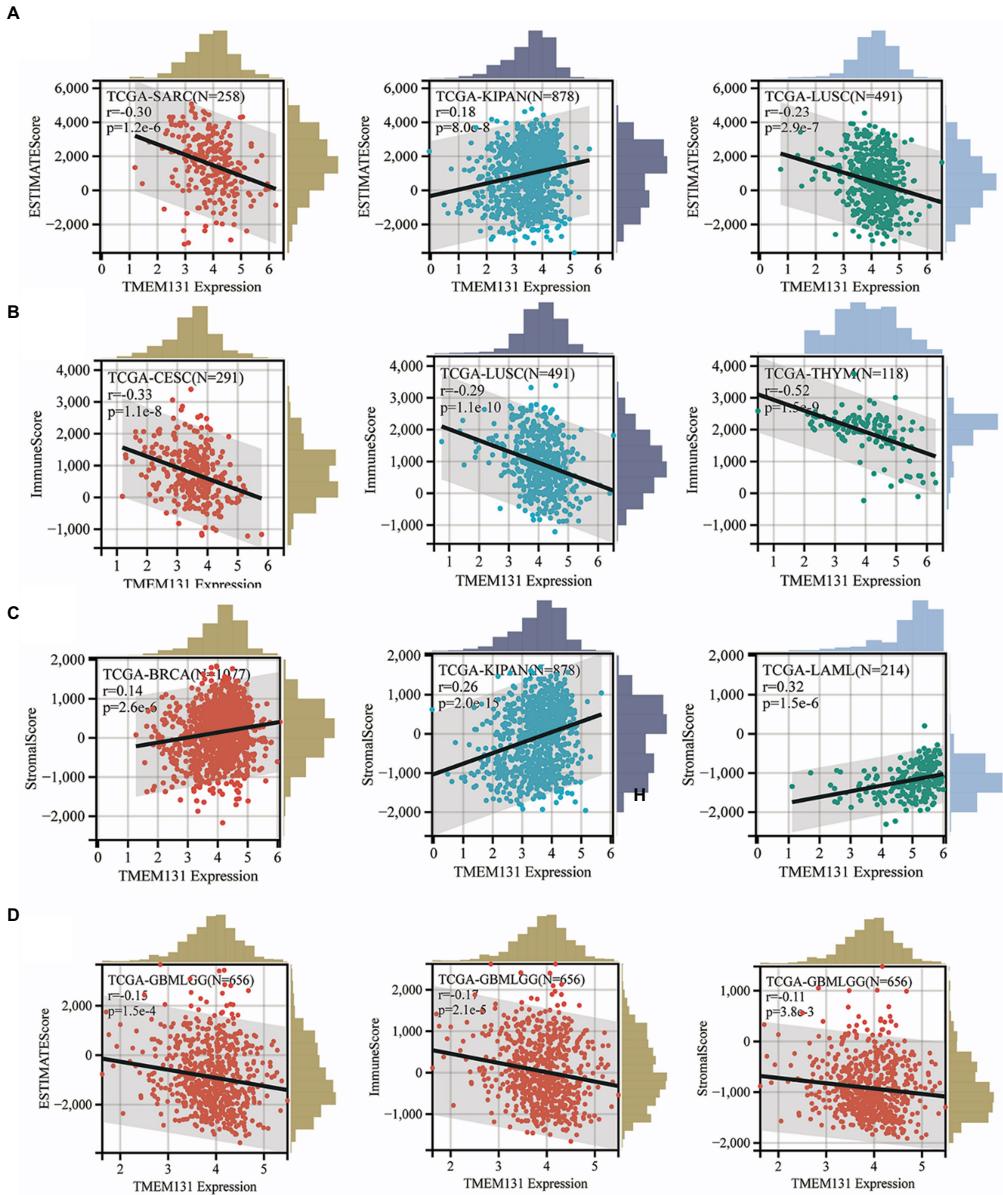
Stromal, immune, and ESTIMATE scores of each tumor patient were calculated according to the expression of TMEM131L. **(A–C)** Top3 scatter plots with the most significant correlation; **(D)** Scatter plots of correlation between TMEM131L and stromal, immunity, and estimation in GBM and LGG, respectively.

In this study, it has been shown that scatter plots of TMEM131L significantly correlated with TOP3 in stroma, immune, and ESTIMATE scores in pan-carcinoma (Figures 9A–C). SARC (*R* = −0.30; *p* = 1.2e−6), Kipan (*R* = 0.18; *p* = 8.0e−8), and LUSC (*R* = −0.23; *p* = 2.9e−7) were associated with the Top 3 of the ESTIMATEScore. CESC (*R* = −0.33; *p* = 1.1e−8), LUSC (*R* = −0.29; *p* = 1.1e−10), and THYM (*R* = −0.52; *p* = 1.5e−9) were associated with the Top 3 of the ImmuneScore. BRCA (*R* = 0.14; *p* = 2.6e−6), Kipan (*R* = 0.26; *p* = 2.0e−15), and LAML (*R* = 0.32; *p* = 1.5e−6) were associated with the Top 3 of the SromalScore. Finally, it was shown that in GCB and LGG, there was a significant negative correlation of TMEM131L scatter plots with ESTIMATEScore, ImmuneScore, and SromalScore with *R* = −0.15; *p* = 1.5e−4, *R* = −0.17; *p* = 2.1e−5, and *R* = −0.11; *p* = 3.8e−3, respectively (Figure 9D).

### Analysis of TMEM131L and immune cell infiltration abundance

**Figure 10:**
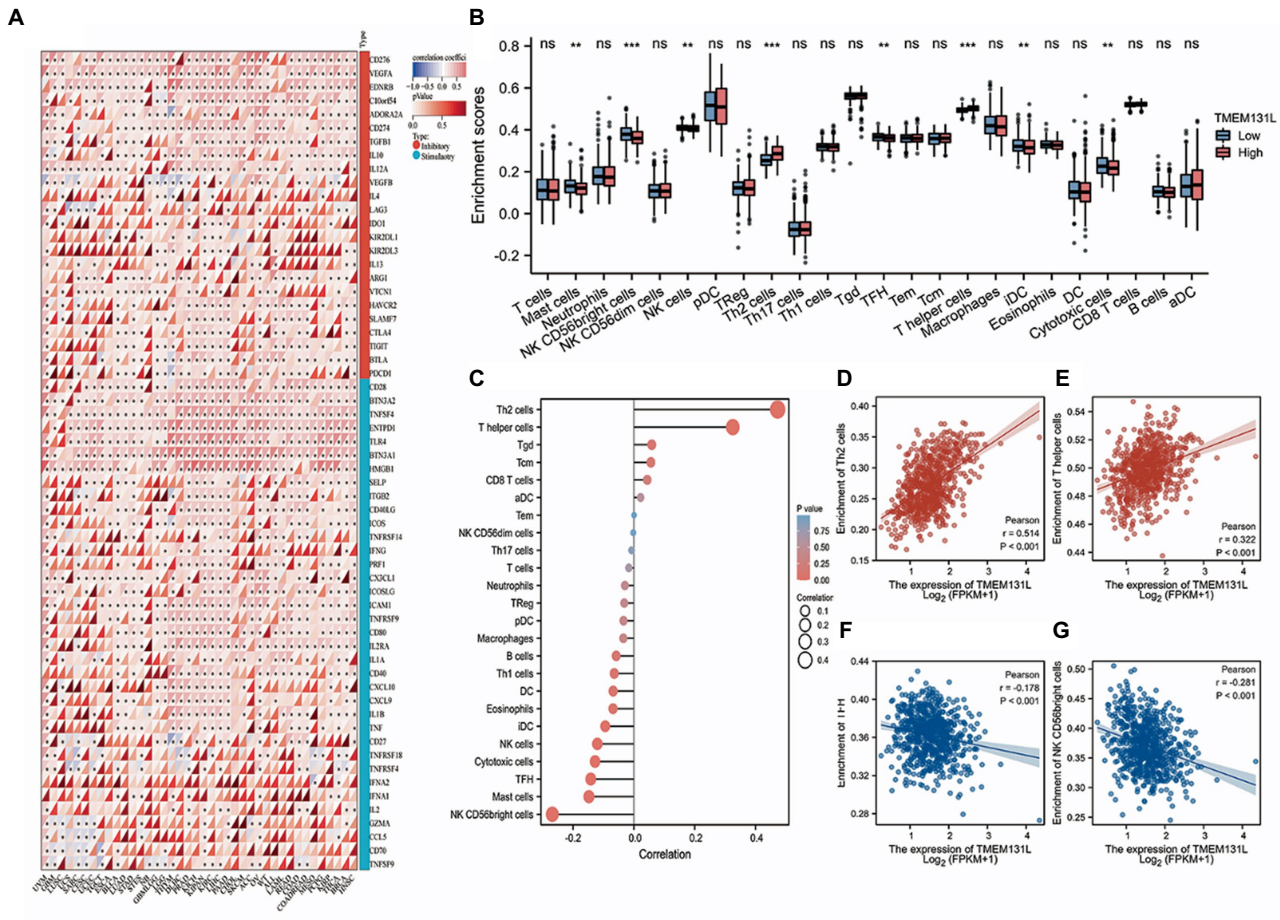
Analysis of immune cell infiltration. **(A)** The expression correlation heat map of TMEM131L and immune checkpoint; **(B,C)** Box plot and lollipop plot CIBERSORT analysis of the relationship between TMEM131L and 22 different types of immune cell infiltration; **(D–G)** CIBERSORT analysis showed that TMEM131L was significantly correlated with 22 kinds of immune cell infiltration.

The correlation between immune infiltration and TMEM131L was the most significant factor. The Pearson correlation coefficient between genes and the levels of immune cell infiltration in each tumor is displayed in a coexpression heat map (Figure 10A). To reflect the immune infiltration of target genes in glioma, an immune infiltration score that significantly associated with TMEM131L expression (Figures 10B,C; Table 3) was identified. Significant correlations were subsequently shown in scatter plots. TMEM131L was negatively correlated with Mast, NK CD56bright cells, TFH, and Cytotomix cells (Figures 10D–G). Helper T cells and Th2 cells were favorably linked to TMEM131L, while NK CD56 bright cells and TFH cells were negatively correlated.

**Table 3:** Correlation between TMEM131L and immune cell infiltration.

	Immune cell	*R* (Pearson)	*p* value (Pearson)
TMEM131L	Th2 cells	0.514	<0.001
	T helper cells	0.322	<0.001
	NK CD56bright cells	−0.281	<0.001
	TFH	−0.178	<0.001
	Mast cells	−0.151	<0.001
	Cytotoxic cells	−0.123	0.001
	NK cells	−0.105	0.006
	Th1 cells	−0.102	0.007
	iDC	−0.101	0.007
	Eosinophils	−0.087	0.022
	DC	−0.070	0.066
	B cells	−0.068	0.074
	pDC	−0.059	0.117
	Tcm	0.059	0.120
	Macrophages	−0.046	0.225
	Neutrophils	−0.043	0.256
	CD8 T cells	0.040	0.288
	Tem	0.022	0.563
	TReg	−0.019	0.615
	Tgd	−0.018	0.627
	Th17 cells	−0.014	0.705
	T cells	−0.013	0.724
	aDC	0.008	0.832
	NK CD56dim cells	−0.005	0.898

### Screening of hub genes related to oxidative stress in TMEM131L

**Figure 11:**
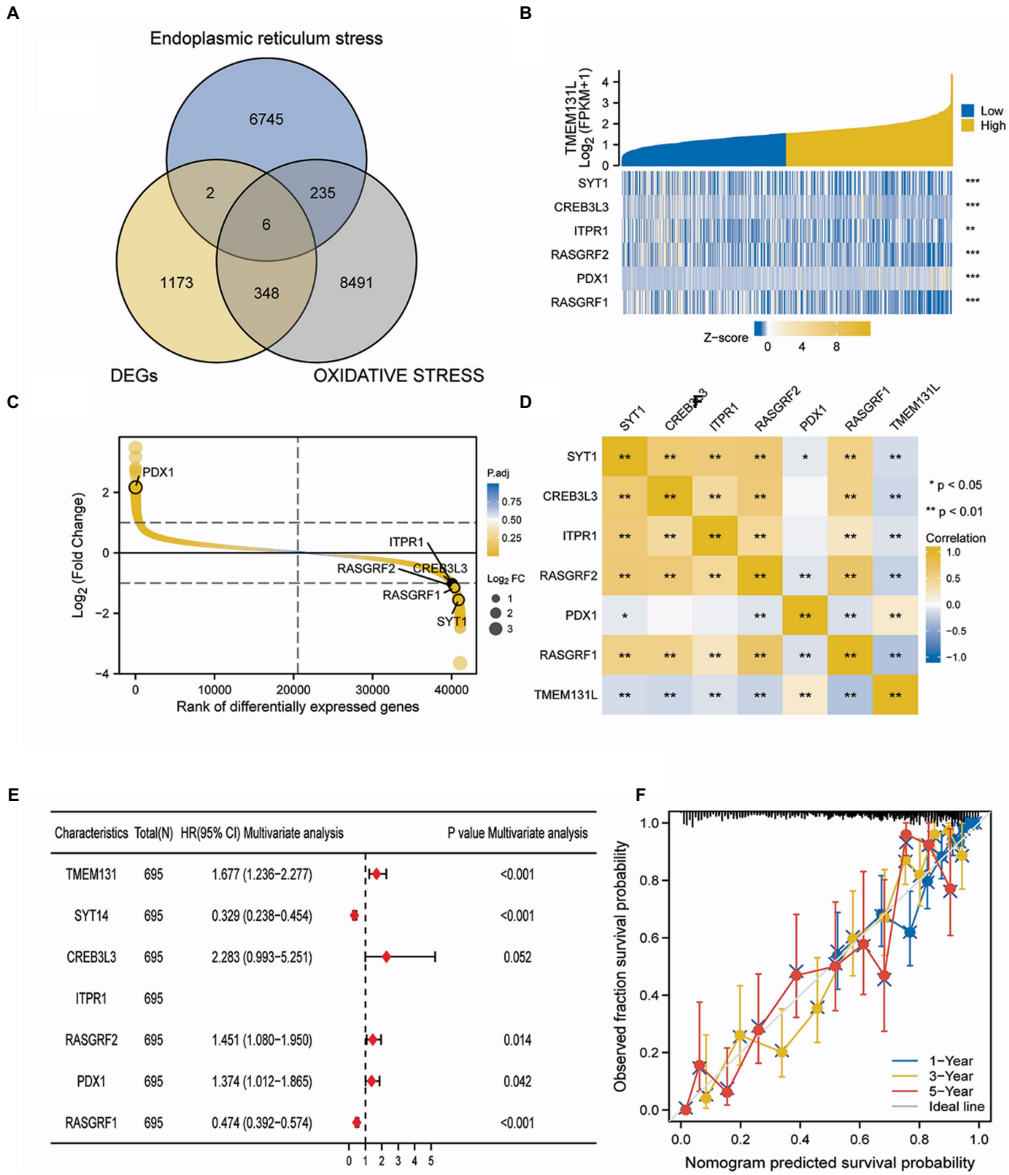
Screening of oxidative stress co-expression genes of TMEM131L. **(A)** Venn diagram of intersection of oxidative stress and endoplasmic reticulum stress phenotypic related genes and TMEM131L co-expressed genes; **(B)** The heat map of the Hub gene expression differences between groups of TMEM131L with high and low expression; **(C,D)** Hub genes and TMEM131L co-expressed in a manner that was depicted by a heat map and differential sequencing map; **(E)** Nomogram of Hub genes for predicting 1-, 3-, and 5-year survival in GBMLGG patients; **(F)** To demonstrate the discrepancy between nomogram prediction and actual survival of Hub genes, calibration curves are used to assess nomogram-predicted 1-, 3-, and 5-year survival.

Using “Oxidative stress” and “Endoplasmic reticulum stress” as search terms, human genes related to phenotypes were collected from the GeneCard database. Meanwhile, the coexpression genes of TMEM131L and TMEM131L were intersected by the Venn diagram, and six oxidative stress-related TMEM131L coexpression genes were obtained. TMEM131L was closely associated with the expression of SYT1, CREB3L3, ITPR1, RASGRF2, PDX1, and RASGRF1 (Figure 11A). Subsequently, the coexpression correlation between Hub genes and TMEM131L was shown by gene expression differential heatmap and coexpression heatmap (Figures 11B–D). Nomograms based on the 1-, 3-, and 5-year survival of patients with GBMLGG were used to evaluate the prediction ability of the screened TMEM131L oxidative stress coexpression genes (Figure 11E). To demonstrate the discrepancy between the nomogram prediction and the Hub gene’s actual survival, the calibration curve’s accuracy was assessed (Figure 11F).

### Construction of a TMEM131L oxidative stress–related prognostic model for glioma

**Figure 12:**
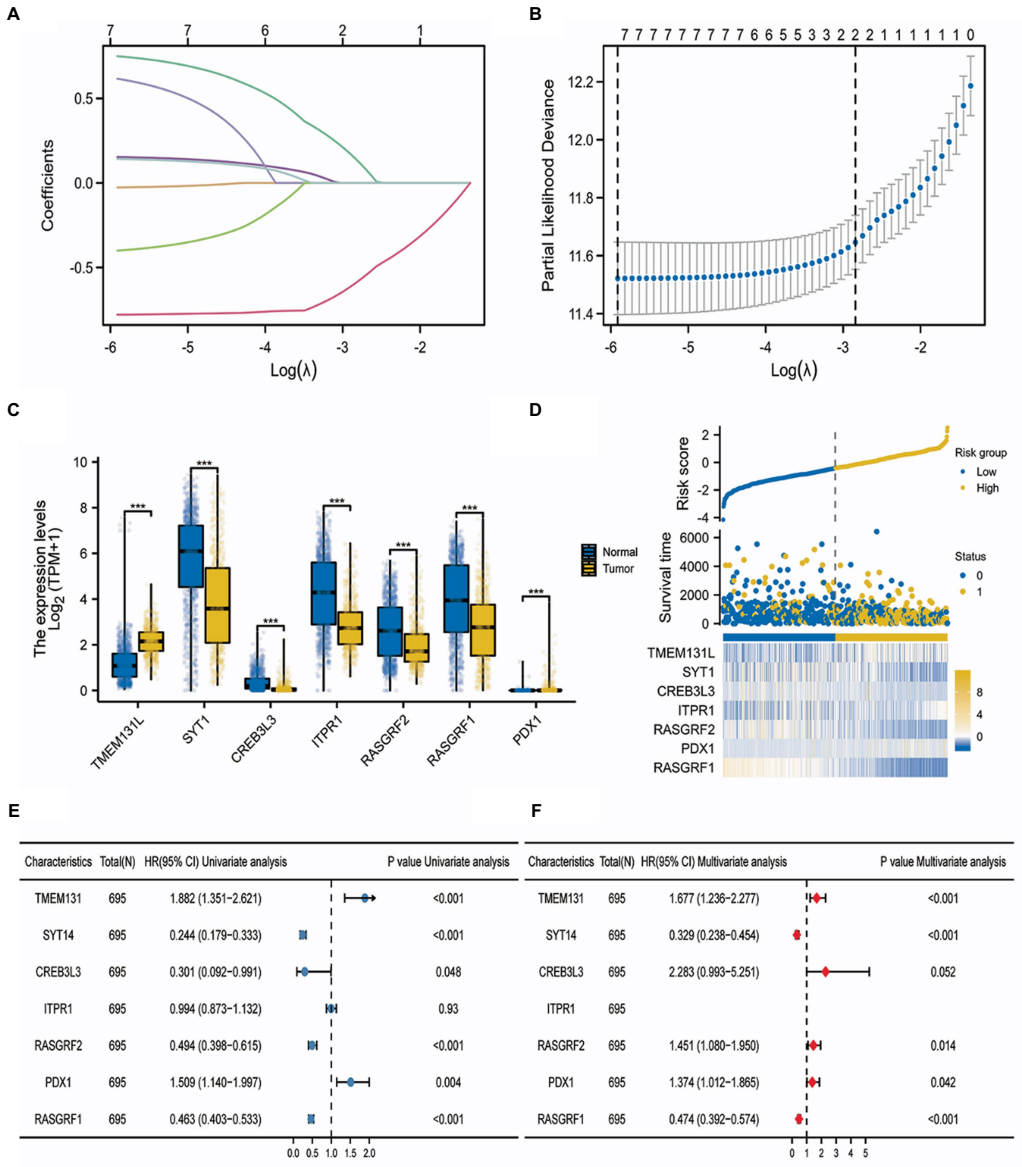
LASSO risk regression model construction. **(A)** LASSO logistic regression algorithm for screening diagnostic markers for lambda visualization; **(B)** min value visualization of diagnostic markers screened by LASSO logistic regression algorithm; **(C)** The expression difference of TMEM131L and Hub gene in glioma; **(D)** Risk curve of risk gene set (top), survival state (middle), and risk heat map (bottom); **(E)** Forest maps of univariate analysis of clinicopathological features of GBM and LGG with risk gene models; and **(F)** Multivariate analysis of clinicopathological features of GBM and LGG with risk gene models.

The LASSO logistic regression algorithm was used to identify six gene signatures from immune-related differential Hub genes between control samples and glioma samples that could be used as prognostic markers for glioma. Figures 12A,B visualizes the Lambda and minimum values of the LASSO logistic regression algorithm (19). Lambda is the weight assigned to the regularized term so that when lambda approaches zero, the model’s loss function will approach the OLS loss function. Meanwhile, the box diagram showed that SYT1, CREB3L3, ITPR1, RASGRF2, and RASGRF1 were significantly underexpressed in tumor tissues (Figure 12C), whereas PDX1 had significant differences in expression but the expression level was low. In parallel, this gene set’s risk heat map (bottom), survival state (middle), and risk curve (top) were created (Figure 12D). A nomogram was created after using univariate and multifactorial Cox to examine the predictive values of risk scores when paired with clinicopathological features of OS (Figures 12E,F).

### Prognostic model using GO and KEGG enrichment analysis

**Figure 13:**
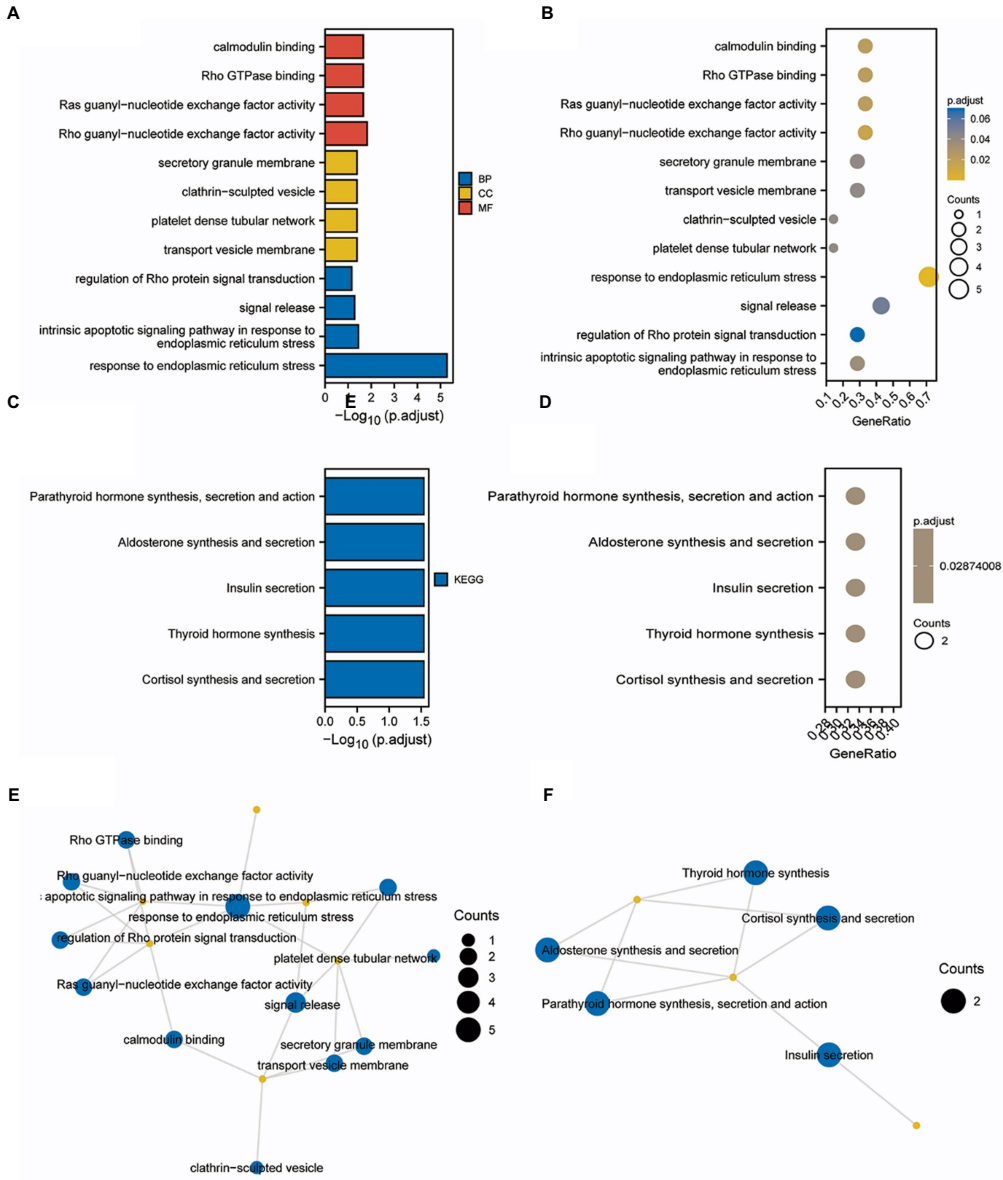
GO/KEGG enrichment analysis of Hub genes related to TMEM131L oxidative stress. **(A)** The length of the column in the GO enrichment analysis bar graph shows the number of enriched genes; **(B)** Analysis of GO enrichment using a bubble map, the significance gradually climbed from yellow to blue while the size of the bubbles reflected the degree of gene enrichment; **(C)** The length of the column in the KEGG enrichment analysis bar graph shows the number of enriched genes; **(D)** According to the bubble map produced by the KEGG enrichment analysis, the size of the bubbles corresponded to the degree of gene enrichment, and the color indicated the significance, which steadily grew from yellow to blue. **(E,F)** Enrichment Term of GO and KEGG enrichment analysis.

**Figure 14:**
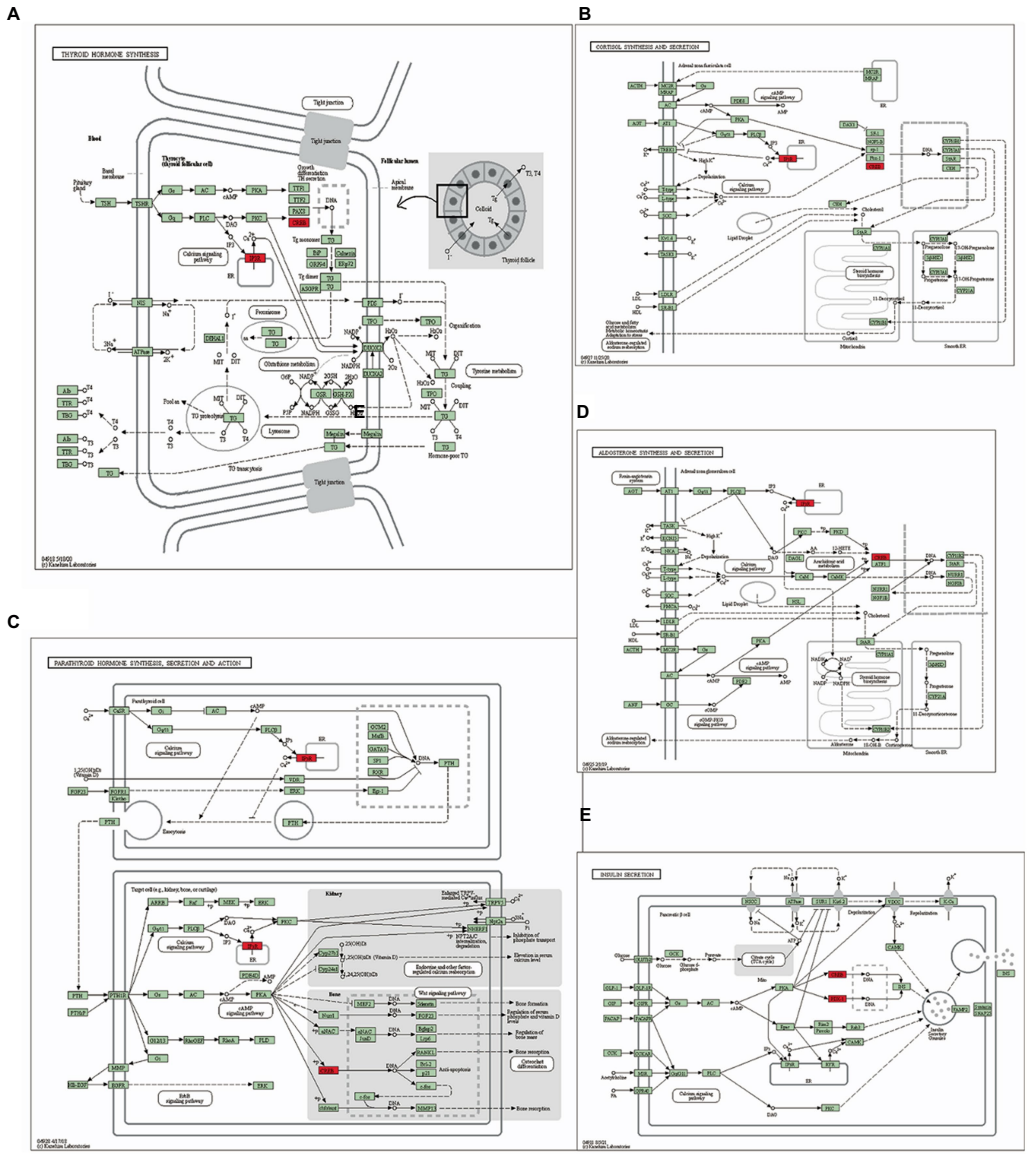
Pathway diagram of KEGG enrichment analysis. **(A)** Thyroid hormone synthesis, **(B)** Cortisol synthesis and secretion, **(C)** Parathyroid hormone synthesis secretion and action, **(D)** Aldosterone synthesis and secretion, and **(E)** Insulin secretion.

Gene ontology and KEGG enrichment analysis of the TMEM131L oxidative stress-associated GBMLGG prognostic Hub gene was performed, and it was found that the gene’s molecular function (MF) was enriched in the function of amino acid metabolism (20, 21). The cellular components were enriched in transport vesicle membranes, platelet-dense tubular networks, clathrin and vesicle secretory granules, and regulation of neuronal synapses by membrane neurons. Meanwhile, the above molecules were enriched in the biological process of insulin secretion (Figure 13). Pathway maps for KEGG enrichment analysis included thyroid hormone synthesis (Figure 14A) and cortisol synthesis and secretion (Figure 14B), parathyroid hormone synthesis and secretion (Figure 14C), aldosterone synthesis and secretion (Figure 14D), and insulin secretion (Figure 14E). Gene function and pathway enrichment results suggest that TMEM131L has a significant potential impact on the occurrence and prognosis of GBM and LGG *via* nerve conduction and information transfer (Table 4).

**Table 4:** GO and KEGG enrichment list of TMEM131L co-expressed hub genes.

ONTOLOGY	ID	Description	*p*.adjust	*q* value
BP	GO:0034976	Response to endoplasmic reticulum stress	5.26e−06	2.81e−06
BP	GO:0070059	Intrinsic apoptotic signaling pathway in response to endoplasmic reticulum stress	0.036	0.019
BP	GO:0023061	Signal release	0.052	0.028
BP	GO:0035023	Regulation of Rho protein signal transduction	0.071	0.038
BP	GO:0050796	Regulation of insulin secretion	0.071	0.038
CC	GO:0030658	Transport vesicle membrane	0.042	0.021
CC	GO:0031094	Platelet dense tubular network	0.042	0.021
CC	GO:0060198	Clathrin-sculpted vesicle	0.042	0.021
CC	GO:0030667	Secretory granule membrane	0.042	0.021
CC	GO:0098984	Neuron to neuron synapse	0.042	0.021
MF	GO:0005089	Rho guanyl-nucleotide exchange factor activity	0.015	0.003
MF	GO:0005088	Ras guanyl-nucleotide exchange factor activity	0.022	0.005
MF	GO:0017048	Rho GTPase binding	0.022	0.005
MF	GO:0005516	Calmodulin binding	0.022	0.005
MF	GO:0005085	Guanyl-nucleotide exchange factor activity	0.022	0.005
KEGG	hsa04927	Cortisol synthesis and secretion	0.029	0.020
KEGG	hsa04918	Thyroid hormone synthesis	0.029	0.020
KEGG	hsa04911	Insulin secretion	0.029	0.020
KEGG	hsa04925	Aldosterone synthesis and secretion	0.029	0.020
KEGG	hsa04928	Parathyroid hormone synthesis, secretion and action	0.029	0.020

### TMEM131L gene set enrichment analysis

**Figure 15:**
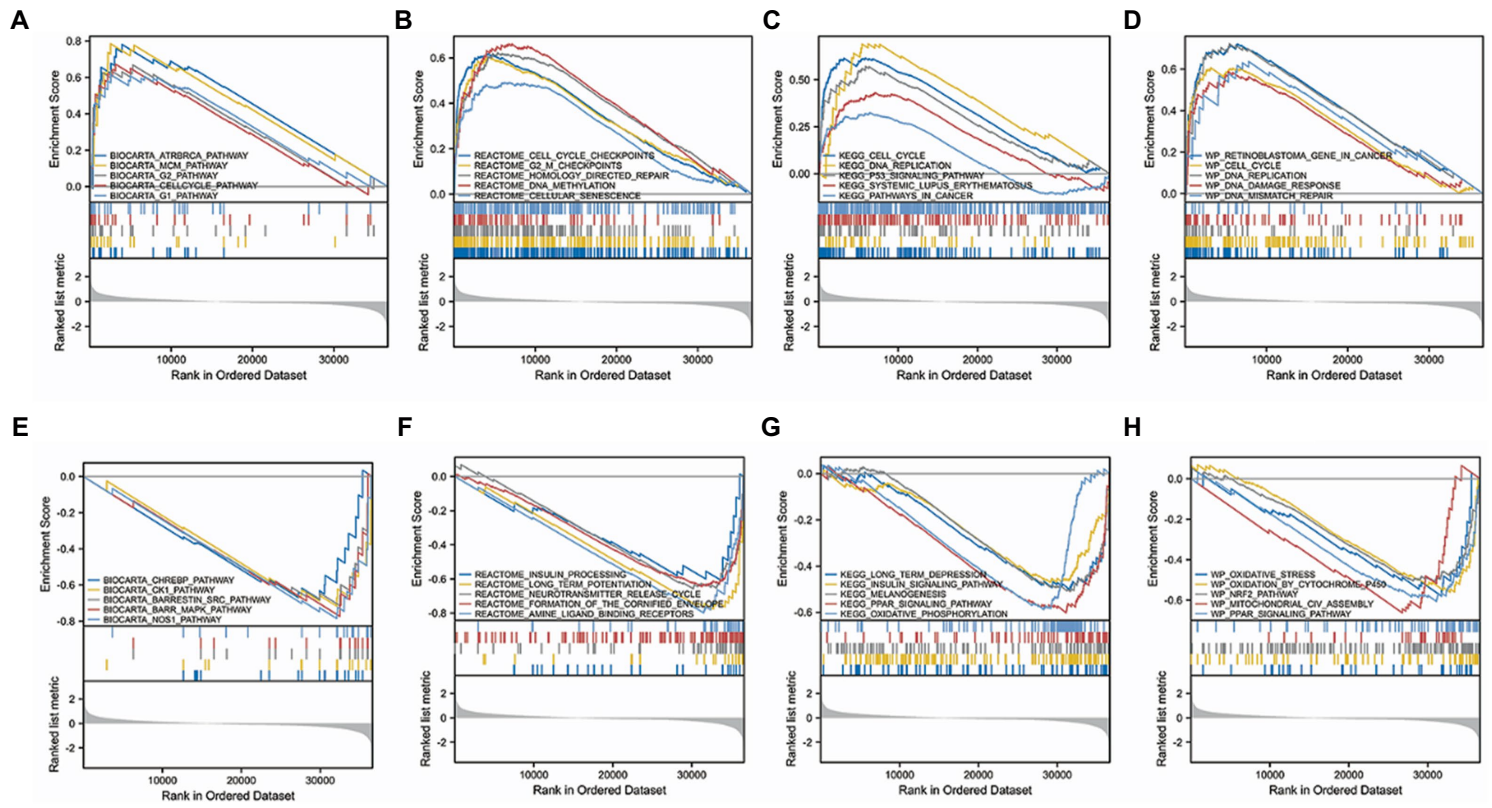
GSEA of TMEM131L. GSEA results of differential TMEM131L expression. Enrichment of TMEM131L with high **(A–D)** and low **(E–H)** expression in BIOCARTA, REACTOME, KEGG, and WP.

Gliomas are malignant solid tumors that form blood vessels and occur primarily in the central nervous system. Gliomas are thought to be highly proliferative and metastatic and resistant to treatment because of the presence of the brain barrier. The computational method, GSEA, was used to determine the statistical significance of a preferentially defined set of genes and to determine the presence of any consistent differences between the two biological states. In this study, thousands of permutations of each gene combination were performed. As a phenotypic marker, TMEM131L expression level was employed. Genome C2. CP. V7.2. Symbols were retrieved from the molecular marker database. GMT and GSEA 4.0.3 were used to analyze the potential enrichment pathways. To divide the enrichment pathway into two phenotypes, the normalized enrichment score (Ness), *p* value, and FDR Q value of GSEA were calculated. In this investigation, significantly enriched genomes were those with *p* < 0.05 and FDR *Q* < 0.25. According to studies, carcinogenic stimuli, increased metabolic activity, and mitochondrial malfunction contribute to cancer cells having higher levels of ROS than normal cells. BIOCARTA database TMEM131L high expression was enriched in the CELLCYCLE pathway of ATRBRCA, MCM, G2, and G1 (Figure 15A), whereas TMEM131L low expression was enriched in the CHREBP, CK1, SRC, BARR-MAPK, and NOS1 pathways (Figure 15E). The REACTOME database high expression of TMEM131L was enriched at cell cycle checkpoints, G2M checkpoints, homology-directed repair, DNA methylation, and cell senescence (Figure 15B), and low expression of TMEM131L was enriched in insulin processing, long-term potentiation, neurotransmitter release cycles, envelope formation, and amine ligand-binding receptors (Figure 15F). The high expression of TMEM131L was enriched in cancer-related molecular signaling pathways, long-term depression, insulin signaling pathway, melanin production (Figure 15C), and the low expression of TMEM131L was enriched in the PPAR signaling pathway, systemic lupus erythematosus, and oxidative phosphorylation route (Figure 15G). The high expression of TMEM131L in the WP database was enriched in DNA repair, mutation, damage, and other related pathways (Figure 15D). Low expression of TMEM131L was enriched in PPAR, NRF2, and oxidative stress (Figure 15H). The elevated expression of TMEM131L may influence the development of glioma by changing the oxidative stress state, according to the aforementioned GSEA (Table 5).

**Table 5:** GSEA significant enrichment results for TMEM131L expression in TCGA-GCBLGG dataset.

Description	ES	NES	*p*. adjust	*q* values
BIOCARTA_ATRBRCA_PATHWAY	0.781	2.244	0.022	0.016
BIOCARTA_MCM_PATHWAY	0.785	2.178	0.022	0.016
BIOCARTA_PGC1A_PATHWAY	−0.829	−2.043	0.022	0.016
BIOCARTA_NOS1_PATHWAY	−0.786	−2.089	0.022	0.016
KEGG_CELL_CYCLE	0.615	2.509	0.022	0.016
KEGG_DNA_REPLICATION	0.691	2.260	0.022	0.016
KEGG_ECM_RECEPTOR_INTERACTION	0.565	2.171	0.022	0.016
KEGG_P53_SIGNALING_PATHWAY	0.571	2.103	0.022	0.016
KEGG_HOMOLOGOUS_RECOMBINATION	0.681	2.096	0.022	0.016
KEGG_SMALL_CELL_LUNG_CANCER	0.525	2.026	0.022	0.016
WP_RETINOBLASTOMA_GENE_IN_CANCER	0.721	2.806	0.022	0.016
WP_DNA_IRDAMAGE_AND_CELLULAR_RESPONSE_VIA_ATR	0.671	2.564	0.022	0.016
WP_CELL_CYCLE	0.607	2.461	0.022	0.016
WP_G1_TO_S_CELL_CYCLE_CONTROL	0.671	2.445	0.022	0.016
WP_DNA_REPLICATION	0.723	2.397	0.022	0.016
WP_GASTRIC_CANCER_NETWORK_1	0.753	2.330	0.022	0.016
WP_MIRNA_TARGETS_IN_ECM_AND_MEMBRANE_RECEPTORS	0.692	2.290	0.022	0.016
WP_GASTRIC_CANCER_NETWORK_2	0.725	2.270	0.022	0.016
WP_DNA_IRDOUBLE_STRAND_BREAKS_DSBS_AND_CELLULAR_RESPONSE_VIA_ATM	0.619	2.205	0.022	0.016
WP_INFLAMMATORY_RESPONSE_PATHWAY	0.702	2.197	0.022	0.016
REACTOME_CELL_CYCLE_CHECKPOINTS	0.618	2.797	0.027	0.019
REACTOME_MITOTIC_SPINDLE_CHECKPOINT	0.671	2.687	0.022	0.016
REACTOME_ACTIVATION_OF_ATR_IN_RESPONSE_TO_REPLICATION_STRESS	0.807	2.628	0.022	0.016
REACTOME_COLLAGEN_DEGRADATION	0.711	2.589	0.022	0.016
REACTOME_G2_M_CHECKPOINTS	0.606	2.560	0.022	0.016
REACTOME_HOMOLOGY_DIRECTED_REPAIR	0.623	2.549	0.022	0.016
REACTOME_DEPOSITION_OF_NEW_CENPA_CONTAINING_NUCLEOSOMES_AT_THE_CENTROMERE	0.679	2.539	0.022	0.016
REACTOME_MITOTIC_G1_PHASE_AND_G1_S_TRANSITION	0.610	2.537	0.022	0.016
REACTOME_DNA_DOUBLE_STRAND_BREAK_REPAIR	0.598	2.530	0.022	0.016
REACTOME_CONDENSATION_OF_PROPHASE_CHROMOSOMES	0.664	2.473	0.022	0.016

### TMEM131L promotes the development of glioma

**Figure 16:**
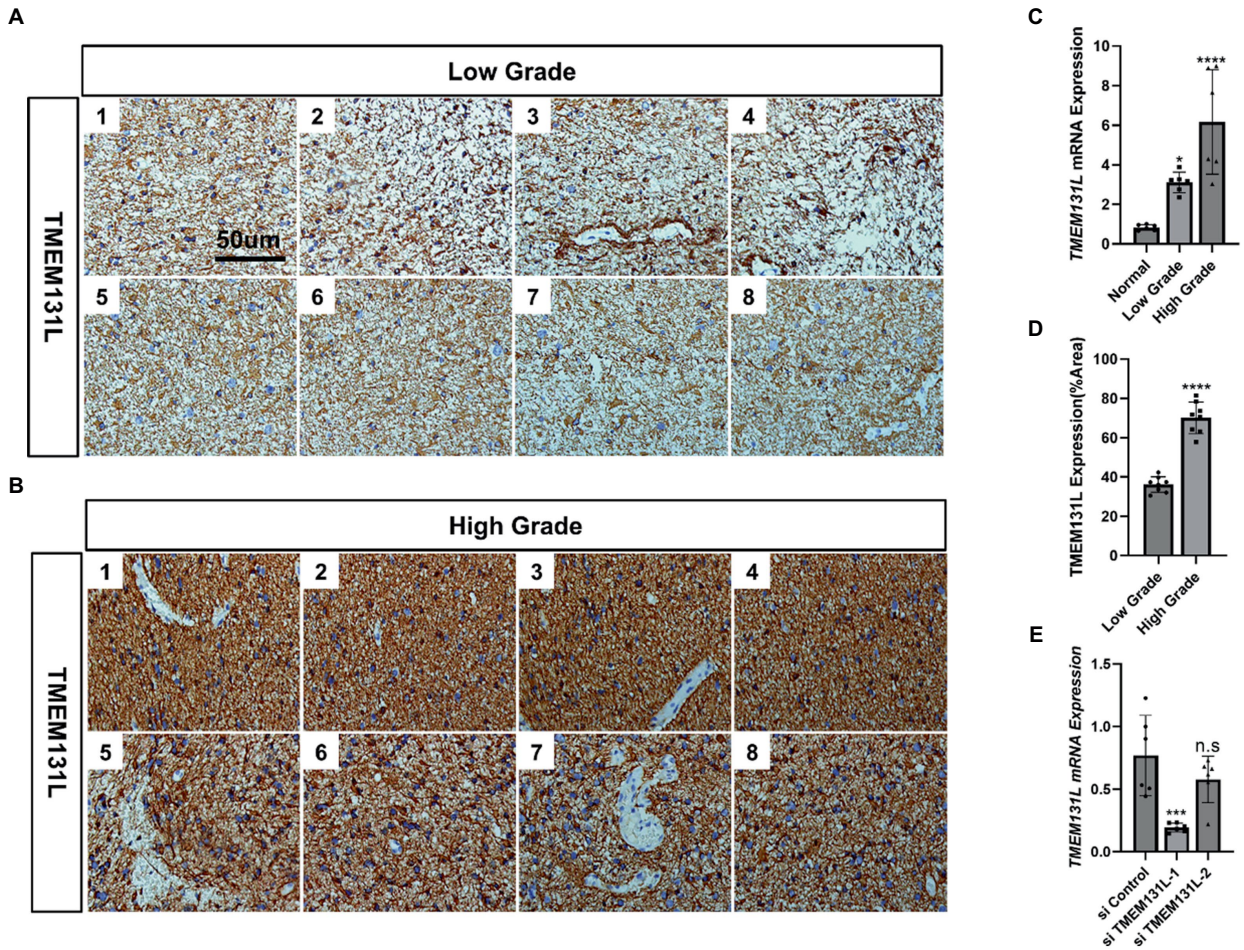
TMEM131L is elevated in glioma. **(A)** Expression of TMEM131L in low-grade gliomas (*n* = 8); **(B)** Expression of TMEM131L in high-grade gliomas (*n* = 8); **(C,D)** The mRNA and protein expression of TMEM131L in low-grade and high-grade gliomas was quantified (*n* = 6); **(E)** Detection and quantification of interference efficiency of si-TMEM131L-1 and si-TMEM131L-2 (*n* = 6). n.s > 0.05, ^****^p ≤ 0.0001.

**Figure 17:**
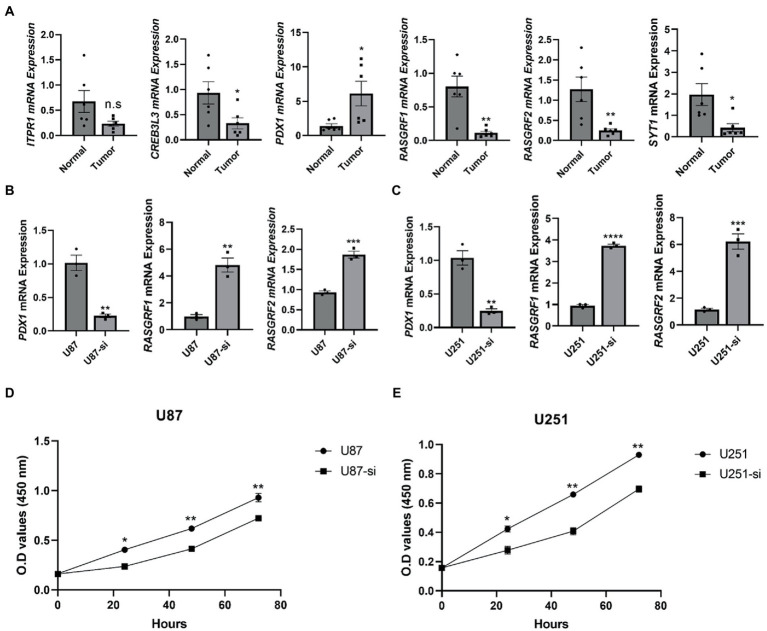
Imbalance of oxidative stress in glioma tissue compared to normal tissue. **(A)** The mRNA levels of *ITPR1*, *CREB3L3*, *PDX1*, *RASGRF1*, *RASGRF2*, and SYT1 were measured and quantified in normal and tumor tissues (*n* = 6); **(B)** The mRNA levels of *PDX1*, *RASGRF1*, and *RASGRF2* were measured and quantified in U87 cell line after interfering with the expression of *TMEM131L* (*n* = 3); **(C)** The mRNA levels of *PDX1*, *RASGRF1*, and *RASGRF2* were measured and quantified in U251 cell line after interfering with the expression of *TMEM131L* (*n* = 3); **(D)** Detection of cell viability after interference with *TMEM131L* expression in the U87 cell line (*n* = 3); **(E)** Detection of cell viability after interference with *TMEM131L* expression in the U251 cell line (*n* = 3). n.s > 0.05, ^*^p ≤ 0.05, ^**^p ≤ 0.01, ^***^p ≤ 0.001, and ^****^p ≤ 0.0001.

**Figure 18:**
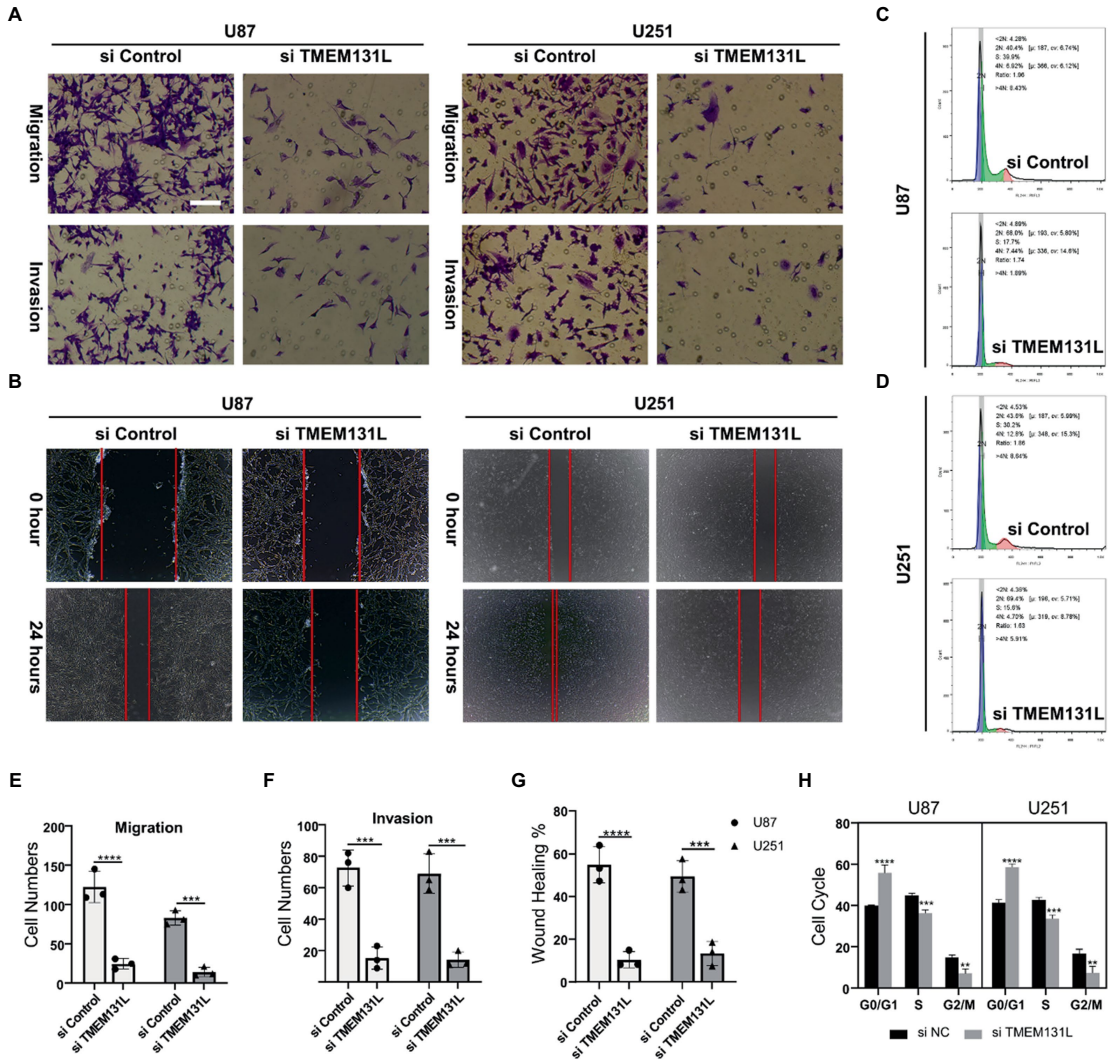
Decreased migration and invasive ability of U87 and U251 after interference with TMEM131L. **(A,E,F)** After interference with *TMEM131L*, the migratory and invasive capacity of U87 and U251 was decreased and cell numbers were quantified (*n* = 3); **(B,G)** After interfering with *TMEM131L*, the invasive ability of U87 and U251 was decreased and quantified for wounds (*n* = 3); **(C,D,H)** Prolonged cell cycle of U87 and U251 after interference with *TMEM131L* expression (*n* = 3). ^**^p ≤ 0.01, ^***^p ≤ 0.001, and ^****^p ≤ 0.0001.

Using immunohistochemistry (IHC) and qRT-PCR, the expression of TMEM131L in various grades of glioma was analyzed. The results revealed that messenger RNA (mRNA) and protein expression of TMEM131L increased with glioma grade (Figures 16A–D). Based on these results, a small interference RNA (siRNA) was designed for two different interference targets and transfected in the U87 cell line. The results of polymerase chain reaction (PCR) showed that the interference efficiency of si-TMEM131L-1 was the highest (Figure 16E). Interestingly, the imbalance of oxidative stress-related pathways was also exhibited in glioma tissues, and the expression of *SYT1*, *CREB3L3*, *ITPR1*, *RASGRF1*, *RASGRF2*, and *PDX1* were significantly different from that of para-cancerous tissues (Figure 17A). Subsequently, we used siRNA to interfere with the expression of TMEM131L in U87 and U251 after which we examined the expression of three of them, PDX1, RASGRF1, and RASGRF2, which are genes related to oxidative stress. We found that after interfering with TMEM131L expression, a balance of oxidative stress was exhibited in U87 and U251 cell lines (Figures 17B,C), along with a significant decrease in cell viability of U87 and U251 (Figures 17D,E). Finally, after the expression of TMEM131L was reduced using si-TMEM131L in the U87 and U251 cell lines, a significant decrease in the migration and invasion ability of the U87 and U251 cell lines was found (Figures 18A,E,F). The wound healing test also revealed the nonhealing of wounds (Figures 18B,G), the cell cycle of U87 and U251 were also prolonged (Figures 18C,D,H).

## Discussion

Gliomas are the most aggressive of all brain tumors. The prognosis for gliomas has been improved *via* various methods. However, the prognosis is still considered poor. In this study, the focus is on glioma prognostic genes. In glioma tissues, it was discovered that TMEM131L was substantially expressed (22). The brain is very vulnerable to ischemia and hypoxia because it is associated with oxidative stress (23). This process leads to oxidative stress, a process that involves multiple mechanisms to satisfy the brain’s need for antioxidant activity during ischemia. For example, nicotinamide adenine dinucleotide phosphate (NADPH) (23) is a major reducing agent that neutralizes oxidants. PPP requires adequate glucose supply to produce NADPH (24, 25), and TP53-induced glycolysis and apoptosis regulators enable neurons to respond to oxidative stress by increasing NADPH (26). The imbalance of the cell environment and disruption of the pathways involved in cell survival are caused by the free radicals, and ROS produced by the oxidation of the antioxidant defense system (27), proliferation, and apoptosis promote the survival of tumor cells, induce cell proliferation, protect cells from apoptosis, and promote the occurrence of various cancers (25, 28). As cancer cells are under constant oxidative stress, they become well adapted to it through several mechanisms that not only activate the reactive oxygen scavenging system but also inhibit apoptosis (29).

In this investigation, TMEM131L protein has been identified as a new regulator influencing the immune microenvironment and prognosis for gliomas. It has been demonstrated that TMEM131L is a new regulator of thymocyte proliferation and Wnt signaling inhibitor. The molecular mechanism mediated by the short hairpin RNA of TMEM131L may lead to thymocyte hyperproliferation and defective development of CD34(+) hematopoietic progenitors within the thymus. This may inhibit classical Wnt/β-catenin signaling by affecting the LRP6 coreceptor level. Here, a xenogeneic Drosophila transgenic model was used to investigate the evolutionarily conserved function of TMEM131L.

Further clinical correlation analysis and survival analysis confirmed the significance of TMEM131L in the diagnosis and prognosis of GBM and LGG. The AUC of the ROC curve reflected the diagnostic efficacy of TMEM131L for histological type, IDH status, primary treatment outcome, age, and 1P/19Q codeletion. Time-dependent ROC curves and K-M survival analyses at 1, 3, and 5 years showed that TMEM131L could be used as a novel molecular marker of poor prognosis in patients with GCB and those with LGG with OS, PFI, and DSS. Through correlation analysis of TMEM131L and expression of DNA methylation sites, potential sites of TMEM131L methylation were screened. TMEM131L was significantly correlated with the stromal, immune, and estimated fraction of GBM and IgG, suggesting that TMEM131L may affect the immune microenvironment of glioma. The correlation between TMEM131L and immune checkpoint was further analyzed. Twenty-two different forms of immune cell infiltration were strongly correlated with TMEM131L, according to a CIBERSORT study. The relationship between TMEM131L and important genes was examined to understand further the molecular mechanisms through which TMEM131L influences the prognosis of gliomas. The results showed that TMEM131L was positively correlated with ROS1 and Sox2. Thus, the coexpression genes of TMEM131L associated with oxidative stress phenotype were screened. The LASSO regression model was constructed to screen the molecular models related to glioma prognosis, which were composed of SYT1, CREB3L3, ITPR1, RASGRF2, PDX1, and RASGRF1. Nomogram and calibration curves further confirmed that the model composed of the key oxidative stress-related TMEM131L coexpression gene has good stability and potential application value for the poor prognosis of patients with glioma. This study found that TMEM131L can be used as a novel molecular marker to influence the poor prognosis of glioma and possibly influence the immune microenvironment of glioma. A prognostic risk model of TMEM131L-RELATED high-performance patients with glioma was further established and validated. The molecular mechanisms underlying the potential role of this model in influencing the prognosis of patients with glioma were subsequently explored by enrichment analysis. GO and KEGG enrichment analysis of the TMEM131L oxidative stress-associated GBMLGG prognostic Hub gene was performed and it was found that TMEM131L’s MF was enriched in amino acid metabolism. Cell components are closely related to cell transport and regulation of synapses. Meanwhile, it is enriched in the biological process of insulin secretion (30). KEGG enrichment analysis of the potential mechanisms of action is related to various thyroid hormone (31), cortisol synthesis, and secretion (32). The results of gene function and pathway enrichment suggest that TMEM131L has a significant potential influence on the occurrence and prognosis of GBM and LGG through nerve conduction and information transmission. GSEAs based on TMEM131L expression were strongly associated with cell cycle, insulin, steroid hormone, neurotransmitter release cycles and carcinogenic stimuli, increased metabolic activity, and mitochondrial dysfunction (33).

## Data availability statement

The datasets presented in this study can be found in online repositories. The names of the repository/repositories and accession number(s) can be found in the article/supplementary material.

## Ethics statement

The studies involving human participants were reviewed and approved by Ethics Committee of the Soochow University Affiliated Taicang Hospital. The patients/participants provided their written informed consent to participate in this study.

## Author contributions

XC conceived and supervised the study. LS, XZ, H-ZQ, and E-DZ contributed to sample collection, basic experiments, data processing, and integrative analyses. LS and XZ wrote the manuscript. All authors contributed to the article and approved the submitted version.

## Funding

This work was supported by the Wu Jieping Medical Foundation Special Fund for Clinical Research (320.6750.2022-09-39).

## Conflict of interest

The authors declare that the research was conducted in the absence of any commercial or financial relationships that could be construed as a potential conflict of interest.

## Publisher’s note

All claims expressed in this article are solely those of the authors and do not necessarily represent those of their affiliated organizations, or those of the publisher, the editors and the reviewers. Any product that may be evaluated in this article, or claim that may be made by its manufacturer, is not guaranteed or endorsed by the publisher.

## Supplementary material

The Supplementary material for this article can be found online at: https://www.frontiersin.org/articles/10.3389/fneur.2023.1162394/full#supplementary-material

## Footnotes

^1^ https://xenabrowser.net/

^3^ https://portal.gdc.cancer.gov/

^4^ http://timer.cistrome.org/

^5^ http://cis.hku.hk/TISIDB/

^6^ http://cibersort.stanford.edu/
